# Molecular Characterization of Lung Dysplasia Induced by c-Raf-1

**DOI:** 10.1371/journal.pone.0005637

**Published:** 2009-05-20

**Authors:** Astrid Rohrbeck, Volker Steffen Müller, Jürgen Borlak

**Affiliations:** 1 Department of Molecular Medicine and Medical Biotechnology, Fraunhofer Institute of Toxicology and Experimental Medicine, Hannover, Germany; 2 Center for Pharmacology and Toxicology, Hannover Medical School, Hannover, Germany; Baylor College of Medicine, United States of America

## Abstract

**Background:**

Lung cancer is a multistage process with poor prognosis and high morbidity. Importantly, the genetics of dysplasia, a facultative cancer, at the edge of malignant transformation is unknown.

**Methodology/Principal Findings:**

We employed laser microdissection to harvest c-Raf1- induced dysplastic as opposed to transgenic but otherwise morphologically unaltered epithelium and compared findings to non-transgenic lung. We then employed microarrays to search genome wide for gene regulatory networks. A total of 120 and 287 genes were significantly regulated, respectively. Dysplasia was exclusive associated with up-regulation of genes coding for cell growth and proliferation, cell-to-cell signalling and interaction, lipid metabolism, development, and cancer. Likewise, when dysplasia was compared with non-transgenic cells up-regulation of cancer associated genes, tight junction proteins, xenobiotic defence and developmental regulators was observed. Further, in a comparison of the data sets of dysplasia vs transgenic and dysplasia vs non-transgenic 114 genes were regulated in common. We additionally confirmed regulation of some genes by immunohistochemistry and therefore demonstrate good concordance between gene regulation and coded protein.

**Conclusion:**

Our study identified transcriptional networks at successive stages of tumor-development, i.e. from histological unaltered but transgenic lungs to nuclear atypia. Our SP-C/c-raf transgenic mouse model revealed interesting and novel candidate genes and pathways that provide clues on the mechanism forcing respiratory epithelium into dysplasia and subsequently cancer, some of which might also be useful in the molecular imaging and flagging of early stages of disease.

## Introduction

The lung cancer epidemic was the subject of a recent editorial [1]. Indeed, in Europe alone more than 340 000 death per annum are attributable to this cancer, but this disease is by large preventable. There is concluding evidence for tobacco smoke to be the primary cause of lung cancer and more than 4,800 compounds have been identified in the particulate and gas phases of cigarette smoke. Major lung carcinogens in smoke include some of the polycyclic aromatic hydrocarbons, such as benzo*[a]*pyrene, as well as tobacco-specific *N*-nitrosamines. Although a lot of evidence supports the relationship between cigarette smoking and lung cancer, the molecular events associated with early stages of disease remain somewhat elusive. A diverse range of genetic abnormalities are seen in different stages of lung cancer, some of which may be employed as markers of disease progression; others may have a direct role in lung cancer etiology in the context of gene-environment interactions. Characterisation of the cancer genome in lung adenocarcinoma was the subject of a recent study and several reviews [2], [3], [4].

Specifically, eighty percent of the lung cancers are classified as non-small cell lung cancer (NSCLC) whereas the remains 20% are small cell lung cancer (SCLC). Survival of patients diagnosed with non-small-cell lung cancer (NSCLC) is poor; over the last decades the 5-year survival rate remained less than 15%. Survival of lung cancer is, however, strongly associated with the stage of disease at the time of diagnosis. Indeed, 5- year survival rates range from 5% for patients with stage IV lesions to 70% at stage I [5]. Such encouraging outlooks have lead to renewed interest in the search and validation of biomarkers of disease to allow monitoring of individuals at risk for developing lung cancer [6]. Most frequently, diagnosis of lung cancer is at a late stage of disease with its classification being based on morphological appearance and immunohistological methods [7]. An identification of patients at risk for developing cancer at early stages of disease would have a big impact on overall survival. Notably, there has been significant progress in an understanding of the molecular pathogenesis of lung cancer, which includes an identification of genetic and epigenetic events in cancer subgroups [8]. The molecular perturbations associated with early stages of lung cancer are, however, unknown as are the gene regulatory networks forcing dysplastic cells into malignant transformation.

Previous studies had defined atypical adenomatous hyperplasia (AAH) as a preinvasive lesion that progresses from low to high grade dysplasia to invasive adenocarcinoma [9]. Strikingly, the genetic events associated with dysplasia and its progression into invasive carcinomas remains uncertain. Specifically, with low grade dysplasia the architectural and cytological changes of the cell are minimal, while with high grade dysplasia gross cytological irregularities become obvious with larger, columnar cells, cytoplasmic pleomorphism, large hyperchromatic nuclei, uneven chromatin structure and higher mitotic rates. Although these morphological changes are well recognized, the underlying molecular alterations are at best poorly understood. Through the use of refined experimental models new insight into the molecular events associated with dysplasia and its progression into cancers can be obtained.

Here we report findings with a transgenic mouse model, where targeted overexpression of c-RAF to respiratory epithelium resulted in lung cancer development [10]. A serum and lung adenocarcinoma proteome map of the SP-C/c-raf transgenic line was recently published by us [11], [12]. We also published the molecular organisation of the c-raf promoter [13]. Specifically, the raf family (a-raf, b-raf and c-raf) of proteins code for serine/threonine kinases, originally isolated as a viral oncogene contributing to cellular transformation and are one of the best characterized Ras effectors to activate the mitogen-activated protein kinase (MAPK) signaling pathway [14]. RAF directly phosphorylates and activates MEK via two conserved serine residues in the kinase activation loop of MEK [15]. Activated MEK then directly phosphorylates a conserved tyrosine and threonine residue in the kinase activation loop of ERK [16]. The MAPK pathway is deregulated in many human malignancies through aberrant signaling upstream of the protein and by activating mutations of the protein itself, both of which induce a proliferative advantage. Indeed, mutations of the K-ras gene have been identified in up to 30% of lung adenocarcinomas and have been considered as a poor prognostic factor [17] underscoring the important role of this pathway in human lung cancer.

As of today, the genetic events associated with dysplasia are basically unknown. We therefore aimed for an identification of preneoplastic changes in a c-raf-1 lung cancer disease model. In our study, we used predominately 5 month old mice to gain information at an early stage of tumor development where isolated foci of transformed cells in distinct areas of the lung are visible. By use of laser microdissection pressure catapulting (LMPC) dysplastic cells in well defined lesions [18] could be isolated. Dysplastic lesions were than compared with transgenic but otherwise normal cells or tumor cells. We applied gene expression profiling to determine differentially expressed genes as to identify the gene regulatory networks associated with dysplasia. Overall, our study revealed interesting and novel genes and pathways that may contribute to the early stages of lung cancer development many of which are worthy for their exploration as candidate biomarkers and for molecular imaging of early stages of disease.

## Results

### Histological changes

**Figure 1:**
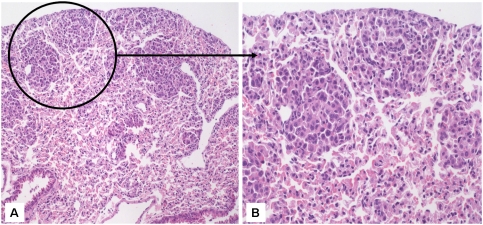
Histological analyses of lung tissue from 5-month-old transgenic mice. Histological analyses of lung tissue from mice transgenic for lung-targeted expression of the cRaf-1 protein. Lung tissue from a 5-month-old SP-C-c-Raf mouse were sectioned at 10 µm, fixed in methanol/acetig acid and stained with H&E. We detected single dysplastic foci, whereas in 10-month-old SP-C-c-Raf mice were multiple foci. A: Overview presentation (magnification: ×50), B: dysplastic foci (magnification: ×200).

Animals at the age of 5 month displayed morphological changes typical for dysplasia. Note, Figure 1 depicts lung tissue from transgenic mice where columnar epithelium, typical for bronchioles with vertically oriented nucleus is replaced by cells displaying horizontal orientation. Cytologically nuclei of different size and unevenly arranged chromatin are observed. Nuclei are hyperchromatic, often with prominent nucleoli. The shapes of the nuclei are irregular and the ratio of nucleus to cytosol area is grossly changed.

### SAM (Significance Analysis of Microarrays)

**Figure 2:**
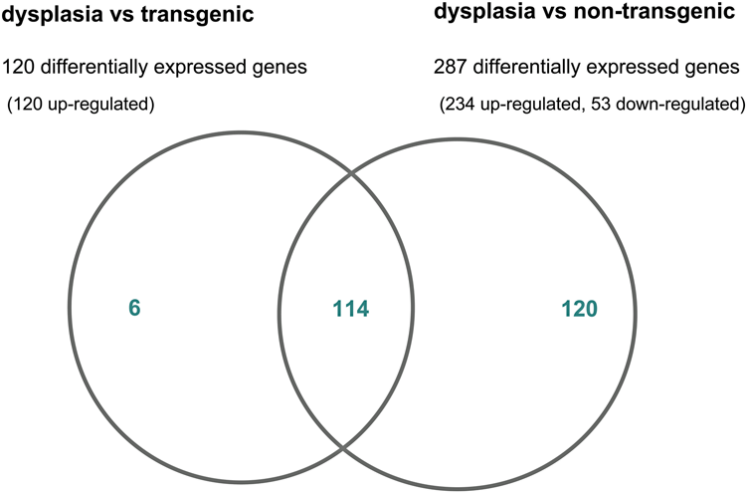
Venn diagram for significantly regulated genes. Venn diagram for significantly up-regulated genes. Comparison of dysplasia and transgenic unaltered lung tissue with non-transgenic samples. 114 genes were found in dysplasia, respectively, which were at least 2-fold differentially expressed (FDR = 0.001).

120 genes were significantly regulated when transgenic but otherwise unaltered cells were compared with dysplastic cells (Supplementary Table S1). Strikingly, dysplasia is exclusively associated with induction of transcript expression. In contrast, when transcript expression in dysplasia was compared with non-transgenic lung tissue 234 up-regulated and 53 down-regulated genes could be determined (Supplementary Table S2). For this comparison, we requested at least 2-fold differentially expressed genes at an estimated false discovery rate of ≤0.001 (Table 1). Notably, transgenicity alone was associated with 16 up-regulated and 2 down-regulated genes (Supplementary Table S3). We searched for differentially expressed genes by comparing the data sets of dysplasia vs transgenic and dysplasia vs non-transgenic. In such a comparison 114 genes were regulated in common (Figure 2).

**Table 1:** Overview of significant differentially expressed Genes (FDR = 0.001, FC ≤2≥).

	non-transgenic	transgenic	dysplasia
**non-transgenic**	0	18	287
**transgenic**	18	0	120
**dysplasia**	287	120	0

Notably, we used this stringent cut-off because we desired a smaller number of genes with very low false positive rates on which to focus our attention. With an estimated false discovery rate of 0.1 we obtained in dysplasia 2352 significantly regulated genes (867 up-regulated and 1485 down-regulated) compared with transgenic but unaltered cells and 3311 genes (1279 up-regulated and 2032 down-regulated) in dysplasia versus non-transgenic cells. Comparison of these data sets resulted in 2207 genes regulated in common in dysplasia (data not shown).

### Principal component analysis (PCA) and hierarchical gene cluster analysis (HCA)

**Figure 3:**
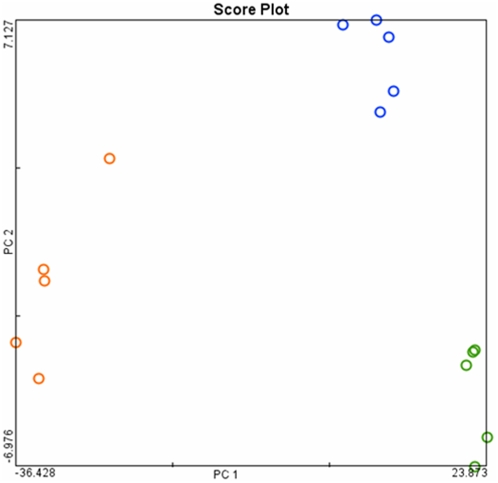
Principal component analysis for gene expression profiles of transformed cells. Principal component analysis of transformed cells from transgenic SP-C/c-raf mouse model in comparison to unaltered lung tissue of transgenic and non-transgenic mice were conducted using the autoscaled method within ArrayTrack. orange; dysplasia; blue, transgenic non-tumor sample; green, non-transgenic samples.

The expression levels were analyzed by the GCOS and ArrayTrack software. We initially examined the data in a 34,000 genes ×15 sample matrix. The PCA classified the data into 3 major groups, namely non-transgenic, transgenic and dysplasia (Figure 3).

**Figure 4:**
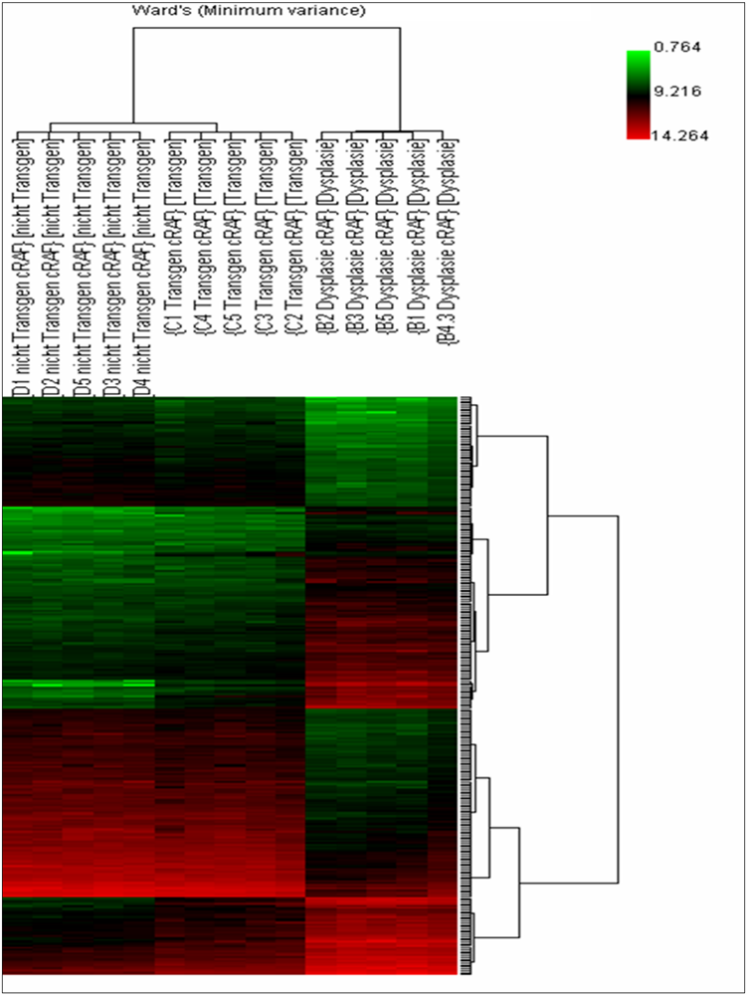
Result of the hierarchical cluster analysis. The normalized data were used for the Ward's Minimum Variance linkage clustering algorithm. A total of 2909 differentially expressed genes (mean channel intensity >100, FDR: 0.1, Bad Flags: 5) were used in the cluster dendogram to obtain a clear segregation of the analyzed groups (dysplasia, transgenic and non-transgenic). The similarity of gene expression profiles among experimental samples is summarized in a dendogram above the cluster, in which the pattern and the length of the branches reflect the relatedness of the samples. Groups (dysplasia, transgenic and non-transgenic) are presented by columns, and genes in rows. Expression values were colour coded with a red green scale. Green, transcript levels below the median; black, equal to the median and red, greater than median.

We also applied hierarchical gene cluster analyses after SAM analysis. The closest pair of expression values of 2909 differentially expressed genes was grouped together. Consequently, the data are organized in a phylogenetic tree in which the branch lengths represent the degree of similarity between the expression values. A clear segregation of the analyzed groups (dysplasia, transgenic but unchanged lung tissue and non-transgenic lung tissue) was obtained (Figure 4). The PCA and HCA grouped data according to their biological state. Correspondingly, gene expression data of transgenic SP-C/c-raf lung were well separated from non-transgenic and dysplastic cells, suggesting a large difference between these groups.

### Pathway analysis of differentially expressed genes

**Figure 5:**
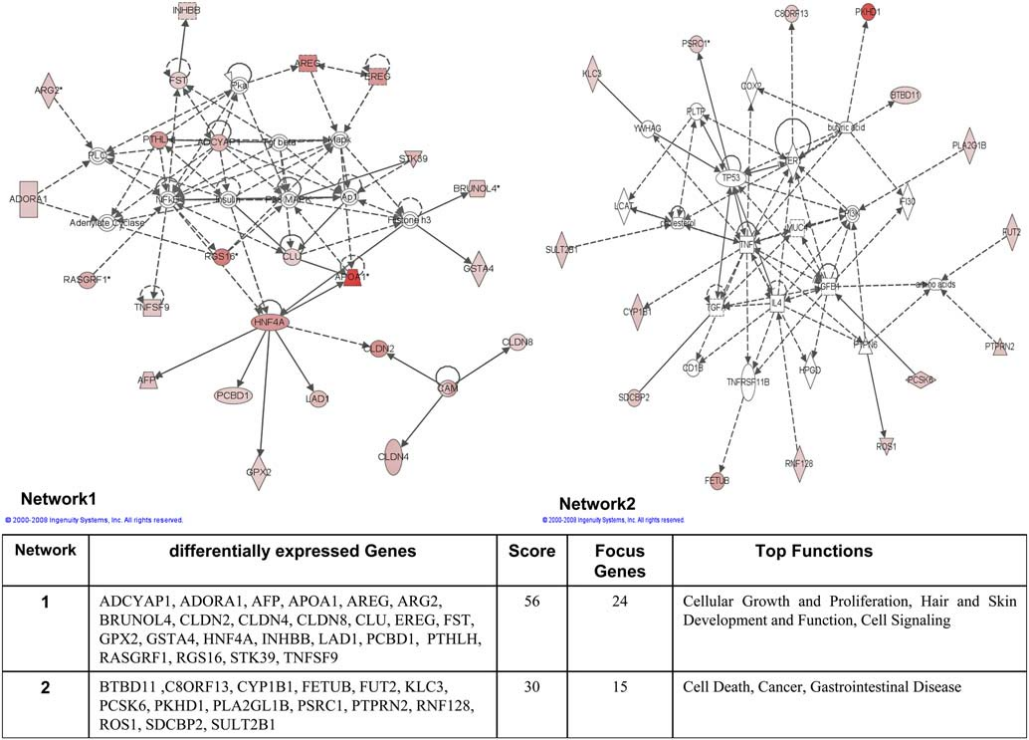
Ingenuity networks: dysplasia versus transgenic mice. Ingenuity networks generated by mapping the focus genes that were differentially expressed between dysplasia and transgenic unaltered lung tissue. Each network is graphically displayed with genes/gene products as nodes (different shapes represent the functional classes of the gene products) and the biological relationships between the nodes as edges (lines). The length of an edge reflects the evidence in the literature supporting that node-to-node relationship. The intensity of the node color indicates the degree of up- (red) or down-regulation (green) of the respective gene. A solid line without arrow indicates protein-protein interaction. Arrows indicate the direction of action (either with or without binding) of one gene to another. IPA networks were generated as follows: Upon uploading of genes and corresponding fold-change expression values (done separately for dysplasia vs transgenic and dysplasia vs non-transgenic differentially expressed genes), each gene identifier was mapped to its corresponding gene object in the IPA Knowledge Base (part of the IPA algorithm). Fold-change expression values were used to signed genes whose expression was differentially regulated; these “focus genes” were overlaid onto a global molecular network contained in the IPA Knowledge Base. Networks of these focus genes were then algorithmically generated based on their connectivity and scored according to the number of focus genes within the network as well as according to the strength of their associations.

**Figure 6:**
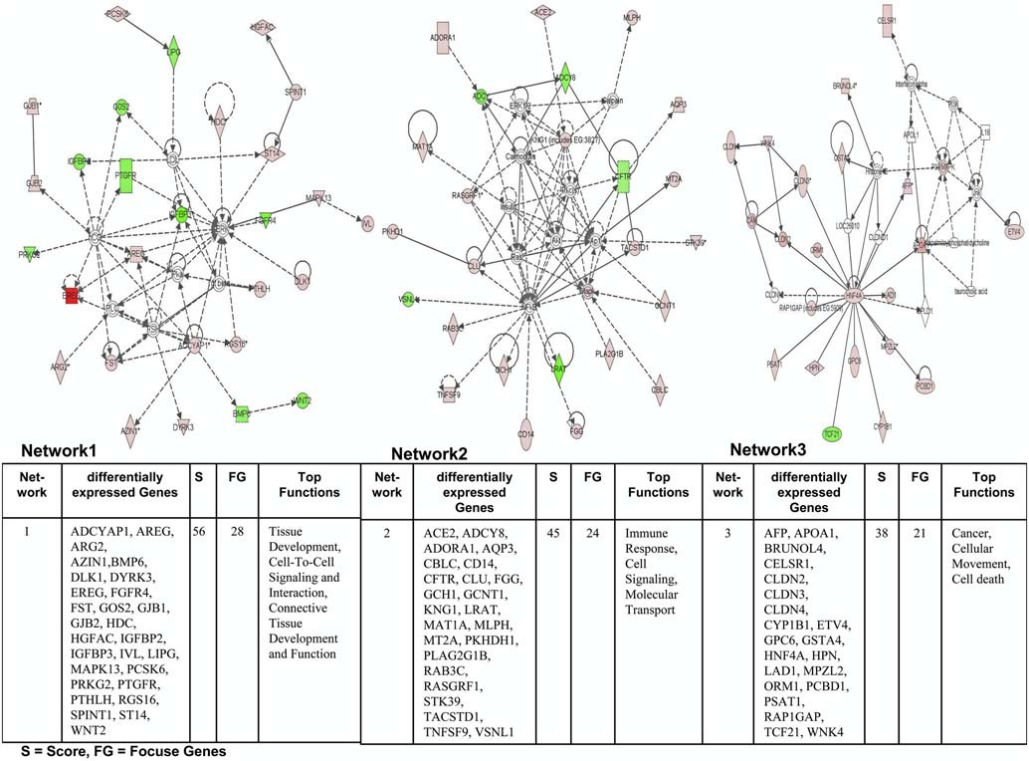
Ingenuity networks: dysplasia versus non-transgenic mice. Ingenuity networks generated by mapping the focus genes that were differentially expressed between dysplasia and non-transgenic unaltered lung tissue (descriptions see Fig. 6).

We employed the Ingenuity Pathways Analysis software and over 70% of regulated genes were mapped to different networks in the IPA database. These networks describe functional relationships among gene products based on findings presented in peer-reviewed biological pathways. Taken collectively, 12 and 16 networks could be defined for the comparison dysplasia vs transgenic and dysplasia vs non-transgenic cells. Based on pathway analysis the top 2 and top 3 networks reached a score of 25 or higher and contained 15 or more genes in the comparison dysplasia vs transgenic (Figure 5) and dysplasia vs non-transgenic cells (Figure 6), respectively. This demonstrates the extensive relationship and interaction between the significantly regulated genes in dysplasia. These networks were associated with the following pathways: cellular growth and proliferation, cell-to-cell signalling and interaction, cancer, lipid metabolism, development, cellular movement amongst others (Supplementary Figure S1). In the following, we describe prominent examples. Cancer cells may produce their own growth factors to stimulate proliferation in neighbouring or parental cells (paracrine vs autocrine loops). We thus searched for genes involved in cellular growth and proliferation and found in the case of dyspalsia 18 genes to be up-regulated ranging between 3.6 to 23.5-fold as compared to transgenic but morphologically unaltered lung tissue. Likewise 36 genes were up-regulated between 4.7 to 482.2-fold when compared to non-transgenic lung tissue. In particular, the Areg (amphiregulin) and Ereg (epiregulin) ligands, both members of the EGF-pathway, were highly significantly over expressed. These molecules play pivotal roles in an activation of the EGF receptor tyrosine kinase to foster proliferation and motility. Overexpressions of these EGF-ligands are observed in a wide variety of human cancers, including breast, prostate and lung cancer.

Cell-to-cell interactions are also important for the regulation of cell proliferation and differentiation. Indeed, expression of cell adhesion molecules is programmed during development to provide positional and migratory information for cells. Disruption of these adhesion events leads to increased cell motility and potential invasiveness trough remodelling of the extra cellular matrix.

We found genes coding for tight junction proteins to be regulated. Identification of the molecular components of the tight junction evidenced that, in addition to their structural functions, these proteins play central roles in regulating cellular proliferation and differentiation. Under normal conditions, tight junctions act to segregate a growth factor within the apical membrane compartment away from its receptor in the basolateral membrane compartment, thereby precluding receptor activation [19]. The disruption of tight junctions in adjacent healthy cells permits the growth factor to bind and activate its receptor, thereby inducing cellular proliferation and migration. In dysplasia the claudins Cldn2, Cldn4 and Cldn8 were significantly up-regulated, their expression ranged from 4.6–13.31-fold and 4.9–122.38-fold, when transgenic but healthy and non-transgenic lung tissue were compared, respectively. Overexpression of these tight junction proteins suggest remodelling of tight junctions.

Next to claudins, we observed up-regulation of gap junction proteins in dysplasia by 11 and 9-fold as well (Gja3, gap junction protein alpha-3 and Gjb4, gap junction protein beta-4). Alteration in gap junctional intercellular communication results in the inability of cells to receive apoptotic, growth suppressing or differentiation signals from their neighbours. Specifically, connexins, a family of 20 trans-membrane proteins in humans, comprise the main subunits of gap junctions; – these specialised clusters of intercellular channels allow adjacent cells to directly share ions and hydrophilic molecules of up to ∼1 KDa in size. Gap junctional intercellular communication is thought to control tissue homeostasis and to coordinate cellular processes such as proliferation, migration and differentiation. Disruption of gap junctional intercellular communication or mutations in connexins is associated with several human diseases. Notably, gap junction expression is often up-regulated in hyperplastic tissues.

Furthermore, lipid metabolism is altered in cancer, including loss of body fat early in tumor growth, induction of hyperlipidemia and changes in a variety of serum lipid and lipoprotein fractions. Thus, cancer patients were found to have higher rates of fat oxidation when compared with healthy individuals with equal weight loss [20], [21]. We examined genes involved in lipid metabolism and found up to 13 genes in dysplasia to be up-regulated ranging from 3.6–28.1-fold and 4.4–148.4-fold when unaltered transgenic and non-transgenic lung tissue was compared with dysplastic cells. In particular Apoa1 (apolipoproteinA-1) the major apoprotein of High Density Lipoprotein (HDL) was significantly over expressed. Apoa1 is a cofactor for Lcat (lecithin-cholesterin-acetyltransferase), which is responsible for the formation of most cholesterol esters in plasma. Apoa1 also promotes efflux of cholesterol, phospholipids, sphingomyelin, sterol and phosphatidylcholin from cells and is involved in transport of cholesterol ester. Recently, it has been observed that the HDL complex is capable of suppressing lymphocyte function, particularly in the host resistance to tumors [22].

In this regard we also found changes in the expression of cell surface glycolipids in dysplastic cells as compared to unaltered transgenic or to non-transgenic lung tissue. For example, Sult2b1 (sulfotransferase family 2b, member 1) was significantly over expressed. This enzyme catalyzes the sulphate conjugation of pregnenolone and cholesterol. Moreover, St8sia6 (St8 alpha-n-acetyl-neuraminide alpha-2,8-sialyltransferase 6), an enzyme which synthesize sialylglycoconjugates of glycolipids was significantly up-regulated, as well. These changes may result in new surface antigens and glycolipids in addition to altered cell-cell and cell-extracellular matrix communication followed by decreased adhesiveness and invasiveness through normal tissue barriers.

Further evidence for changes in glycosylation patterns during transformation of normal cells into malignant ones stems form the up-regulation of B4galt6 (UDP-GAL:Beta-GlcNAc Beta-1,4-Galactosyltransferase, polypeptide 6), Fut2 (Fucosyltransferase 2), Gpc6 (Glypican6) and Orm1 (orosomucoid1) as observed in dysplastic cells. The synthesis of new carbohydrate chains in dysplastic cells are due to activation of glycosyltransferase such as B4galt6, which catalyzes the reaction UDP-galactose and N-acetylglucosamine for the production of galactose beta-1,4-N-acetylglucosamine. Strikingly, these carbohydrates are absent or have low activity in normal cells. Moreover, the secretor enzyme Fut2, an *α*-1,2-fucosyltransferase, is responsible for the transfer of fucose in an *α*-1,2 linkage to form the terminal H type 1 structure. The extra fucosylations that appear on membrane glycoproteins and glycolipids are associated with several pathological processes, such as tumor metastasis, inflammation and bacterial adhesion.

As the expression of glycoproteins is increased in many cancers, it was of no surprise that Orm1 was 11.1-fold overexpressed in dysplastic cells. Orosomucoid 1 belongs to a group of highly glycosylated glycoproteins and appears to function in modulating the activity of the immune system. Furthermore, we found glypican 6 to be induced (9.9-fold). Notably, this protein belongs to a family of glycosylphosphatidylinositol-anchored heparin sulphate proteoglycans. It is known that proteoglycans are high-molecular-weight glycoproteins and interact via their multiple binding domains with many other structural macromolecules. They are bound together with extracellular matrix components and act as cell adhesion factors by promoting organization of actin filaments in the cell cytoskeleton. Proteoglycans have been shown to undergo alterations during malignant transformation resulting in disrupted interaction between the extra cellular matrix and the transformed cells to simplify the invasion into the surrounding tissue.

We further investigated genes involved in developmental processes and found 8 genes to be 3.6–15.4-fold up-regulated in dysplasia as compared to transgenic lung tissue. Notably, 22 genes were 5.4–148.4-fold up-regulated in dysplasia as compared to non-transgenic lung tissue.

In particular Hnf4α (hepatocyte nuclear factor 4-alpha), Foxa3 (forkhead box gene A3) and Foxp2 (forkhead box gene P2) were significantly over expressed in dysplasia (4.5–12.8-fold). These genes codes for proteins that belong to a family of winged-helix/forkhead DNA binding domain transcription factors and are expressed in defined neural, intestinal, and cardiovascular cell types during embryogenesis and differentiation of epithelium. Hnf4α plays a key role in a transcriptional hierarchy and controls the expression of other transcription factors such as Hnf1 (hepatocyte nuclear factor 1) [23]. Indeed, hundreds of genes are targeted by Hnf4α. Since many of those genes contain more than one Hnf4α binding site, these genes can be grouped into several different categories, according to function, such as nutrient transport and metabolism, blood maintenance, immune function, cellular differentiation and growth factors. The best characterized Hnf4α target genes are those involved in lipid transport (e.g., apolipoprotein genes) and glucose metabolism [24]. In this regard Foxa genes code the winged helix/forkhead transcription factor gene family that also includes Foxa1, Foxa2 and Foxa3, as well [25]. Notably, Foxp2 is characterized as transcriptional repressor and is the first Fox gene that is expressed exclusively in the distal epithelium of the lung during pulmonary development [26].

Finally, we searched for expression of oncogenes and found the proto-oncogene Ros1 (v-ros avian ur2 sarcoma virus oncogene homolog 1), which encodes a transmembrane protein with a sequence typical of tyrosine kinases to be 5.8-fold up-regulated in dysplasia. Ectopic expression of this receptor tyrosine kinase Ros1 has been reported in many tumors of the central nervous system and recently in lung and stomach cancers [27] as well. It has been suggested that Ros1 plays a role in the mesenchymal epithelial transition during development of kidney, lung and small intestine [28].

### Quantitative real-time PCR

To confirm the microarray data, expression of 8 genes in dysplasia, transgenic and non-transgenic samples was examined by quantitative real-time PCR using TaqMan Technology. qRT-PCR confirmed that amphiregulin (Areg), epiregulin (Ereg), fetuin beta (Fetub), apolipoprotein A1 (Apoa1), claudin 2 (Cldn2), hepatic nuclear factor 4, alpha (Hnf4α), glutathione S-transferase, alpha 4 (Gsta4) and forkhead box A3 (Foxa3) were up-regulated in dysplasia (Supplementary Figure S2 and S3).

**Figure 7:**
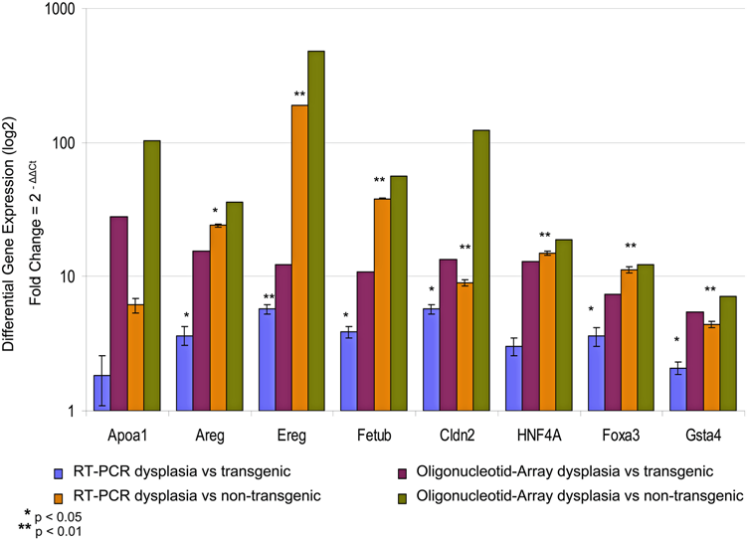
Comparison quantitative RT-PCR and Oligonucleotid-Array. Summary of differential expression of the eight genes verified by quantitative RT-PCR in comparison with Oligonucleotid-Array analysis. The data were analyzed statistically using Student's t-test (*p<0.05; **p<0.01). Error bars indicate standard deviation of five samples and two independent assays for each gene.

The expression data generated by the oligonucleotid array and RT-PCR agreed well, therefore supporting the reliability of the array analysis (Figure 7).

### Immunohistochemistry

**Figure 8:**
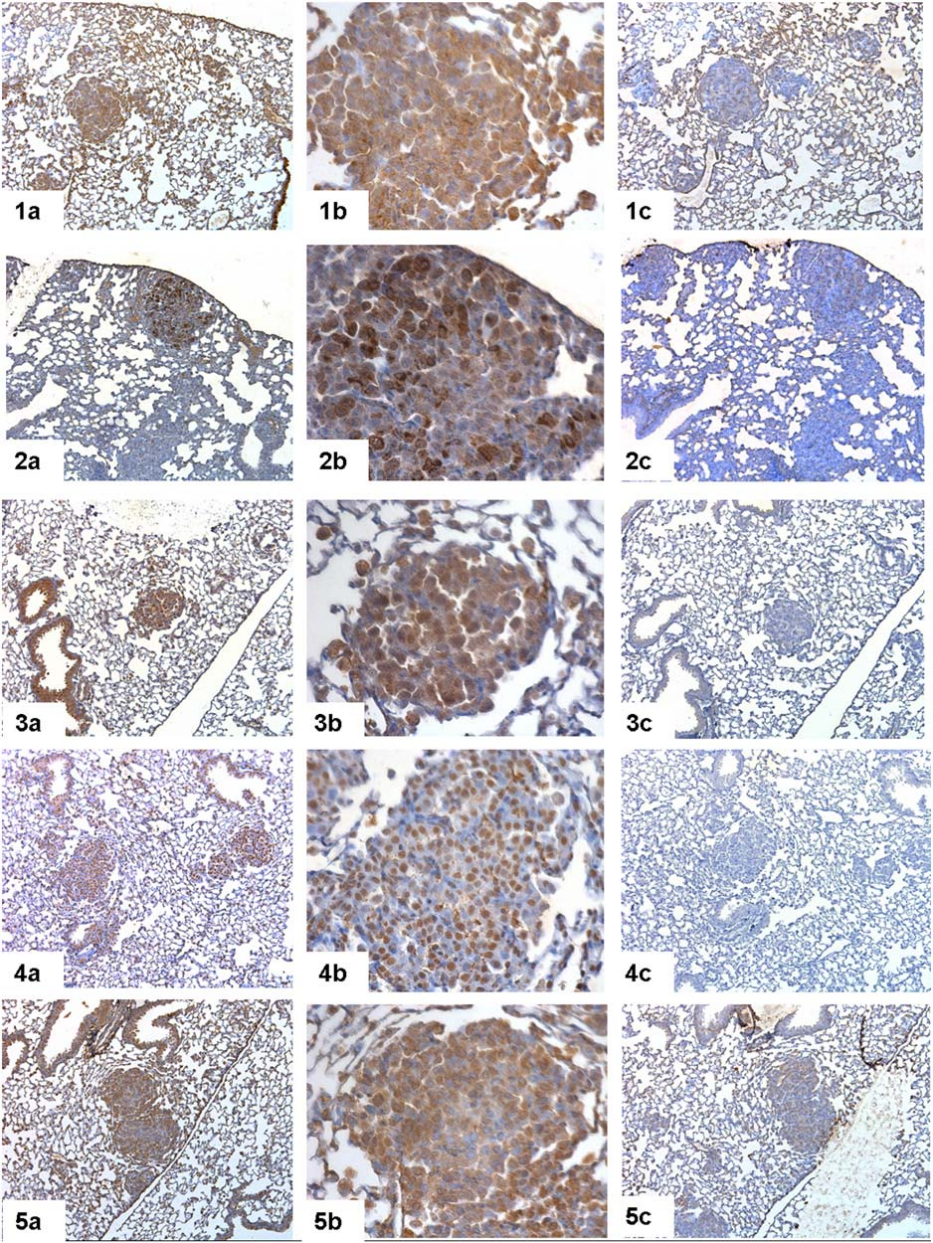
Immunohistochemical staining for dysplasia. Immunohistochemical staining in dysplasia in the presence of primary antibody (a) 10× magnification, b) 40× magnification) and in the presence of primary antibody preincubated with blocking peptide (c). 1 = amphiregulin (AREG), 2 = epiregulin (EREG), 3 = hepatocyte nuclear factor 4 alpha (HNF4α), 4 = forkhead box 3a (FOX3A/HNF3γ), 5 = forkhead box P2 (FOXP2). Strong, mainly cytoplasmic immunoreactivity was found in dysplastic cells, whereas unaltered cells shown only weak positivity using the amphiregulin antibody (1). Epiregulin immunostaining showed a cytoplasmatic pattern of immunoreactivity in dysplastic cells (2). The FOX3A antibody (3) showed strong nuclear positivity in dysplastic cells, while unaltered transgenic cells were negative. The HNF4α antibody showed nuclear immunoreactivity (4). Nuclear positivity was also restricted to dysplastic cells with no staining in unaltered transgenic cells using the FOXP2 antibody (5).

In order to validate array data as well as to add subcellular localization, we performed immunohistochemical staining for some differentially expressed genes. Immunohistochemistry using antibodies towards amphiregulin, epiregulin, hepatocyte nuclear factor 4 alpha, forkhead box A3 and forkhead box P2 showed a consistent difference in immunoreactivity between dysplastic and transgenic but otherwise unaltered cells (Figure 8). For all five up-regulated proteins, patterns of immunoreactivity confirmed the array data and quantitative real-time PCR results.

## Discussion

This study aimed for a better understanding of the genetic events associated with dysplasia in a genetic model of lung cancer induced by overexpression of the c-Raf-1 kinase.

We aimed to determine the regulatory gene networks associated with dysplasia. This enabled identification of candidate genes specifically regulated at the edge of malignant transformation. Based on hierarchical gene cluster analysis and principal-component analysis we were able to distinguish the dysplastic cells from transgenic and non-transgenic lung tissue.

To further validate the microarray data, we employed qRT-PCR of selected genes, and confirmed by immunohistochemistry regulated proteins in dysplastic foci.

Notably, RT-PCR data is suggestive for the microarray to underestimate changes in the gene expression even though both methods supported the general directions of changes. An important finding of our study was that about 10% of the differentially expressed genes regulated by ≥2 fold are already known to be associated with lung cancer.

Specifically, in dysplasia genes coding for nucleic acid metabolism or cell cycle regulation were unchanged but two EGF-ligands, namely amphi- and epiregulin were highly significantly up-regulated. These ligands enable autocrine loops to foster undue EGF-signaling. Notably, Areg (amphiregulin) is a 252-amino acid transmembrane glycoprotein and consist of two major soluble forms of 78 and 84 amino acids, respectively. Areg was originally isolated from conditioned medium of the human breast carcinoma cell line MCF-7 and was found to be a heparin-binding growth factor. Areg promotes neoplastic growth in mammary epithelial cells, fibroblasts, and keratinocytes [29], [30] and was shown to function in an autocrine manner to drive the proliferation of malignantly transformed cells of colon, breast, cervix, prostate, and pancreas [31]. This ligand of the EGF tyrosine kinase is commonly over expressed in cancers of human colon, stomach, breast, and pancreas, in which the level of Areg correlates with tumor progression and poor patient survival [32], [33], [34], [35]. Notably, it was shown in patients with advanced non-small cell lung cancer that the survival time of amphiregulin-negative patients treated with gefitinib was significantly longer than that of amphiregulin-positive patients. Therefore, an increase in serum amphiregulin can be viewed as a predictor of resistance to gefitinib [36]. Moreover, Areg was also reported as an inhibitor of apoptosis in non-small cell lung cancer cell lines [37]. Our data indicate that Areg secreted from dysplastic cells are in an autocrine loop to foster malignant transformation.

A further growth factor strongly induced in dysplasia is Ereg (epiregulin). The gene codes for a transmembrane precursor before being proteolytically cleaved to release a 46-amino-acid activated protein [38]. Ereg is promiscuous, binds and activates the Egfr family member Erbb4 via heterodimeric interactions with Erbb2 [39]. Although Ereg expression is highly correlated with survival from bladder cancer [40], others suggest epiregulin to be important for pancreatic and prostate cancer development [41]. The strongest evidence for a role of epiregulin during tumorigenesis was obtained in Ki-ras-mediated signaling of colon cancer cells [42].

The fact that transformed cells grow faster than unaltered cells is also sustained by our data with regard to the number and strength of up-regulated genes affecting cellular growth. Specifically, the cell surface membrane proteins play an important role in the behaviour of cells to allow for communication with other cells, cell movement and migration, adherence to other cells or structures and recognition by the immune system. Alterations of the plasma membrane in malignant cells may thus be inferred from a variety of properties that characterize their growth and behaviour.

Our study is suggestive for changes in the glycosylation patterns and in cell surface glycolipids. Changes in glycosylation can include the presence of new carbohydrate structures that are not detected in the normal epithelial cells and/or short carbohydrate chains usually masked by larger epitopes. For example, we found St8sia6 to be up-regulated in dysplasia. St8sia6 (alpha-2,8-sialyltransferase VI) belongs to a family of sialyltransferases that synthesize sialylglycoconjugates. The most frequently described aberrant glycosylation in cancer cells include the synthesis of highly branched and heavily sialyted glycans [43]. Because of the negative electric charge of sialic acid at the terminal non-reducing end of glycoprotein oligosaccharides, it plays a key role in mediating biological recognition events, including those responsible for metastasis. Next, we found an overexpression of B4galt6 (UDP-GAL:Beta-GlcNAc Beta-1,4-Galactosyltransferase, polypeptide 6). This enzyme is involved in the production of GlcNAc (galactose beta-1,4-N-acetylglucosamine). GlcNAc is the first sugar residue to be linked to serine or threonine and serves as acceptor for the elongation with further GlcNAc molecules. The resulting branched structure may be elongated by the sequential addition of galactose, fucose and sialic acid [44].

Changes of the glycosylation pattern in dysplasia enables further variability in molecular assembly of membranes and offers a wide range of flexibility in response to cellular environment. Their detection on the cancer cell surface may provide useful diagnostic or prognostic information but until now is incompletely understood and therefore requires further elucidation.

Changes in the expression pattern of cell-adhesion molecules and components of intercellular junctions are related to loss of epithelial organization, proper cell layer and tissue polarity. With regard to adhesion molecules, we found up-regulation of Chl1 (cell adhesion molecule with homology to L1CAM), a member of the L1 gene family of neural cell adhesion molecules, and of several claudins as exemplified by the up-regulation of Cldn2 (claudin2), Cldn4 (claudin4), and Cldn8 (claudin8), which are important components of the tight junctions. Adhesion properties greatly impacts cell-to-cell interaction and growth of cancer cells.

In this regard, the cytoskeleton, which represents a complex of interconnected fibrillar elements, has been determined as an important factor in mediating adhesion-independent and dependent signalling. During morphogenesis, they determine cell shape and polarity, and promote stable cell-cell and cell-matrix adhesions through their interactions with cadherins and integrins, respectively. Surprisingly, in dysplastic cells we did not identify regulation of genes involved in the cytoskeleton.

Instead, we identified changes in cell to cell communication. In our study Gjb3 (gap junction membrane channel protein beta 3) and Gjb4 (gap junction membrane channel protein beta 4) were significantly up-regulated. It was shown that the gap junctional intercellular communication is a form of cell–to-cell signalling thereby mediating the exchange of small molecules between neighbouring cells [45]. The regulation of connexins and gap junctions is a hallmark of carcinogenesis, while their induction in cancer cells leads to reversal of the cancer phenotype, induction of differentiation, and regulation of cell growth [46].

We also focused on genes coding for surfactant lipids in respiratory epithelium. Surfactant is a complex mixture of lipids and proteins that reduces surface tension at the air–liquid interface and prevents alveolar collapse during respiration. Four surfactant proteins (SP) with unique properties have been identified. SP-A and SP-D are relatively hydrophilic proteins and contribute to innate defence of the lung and surfactant homeostasis. SP-B and SP-C are hydrophobic proteins that enhance surface-active properties of surfactant phospholipid films [47]. In dysplastic epithelium we could not detect regulated genes coding for surfactant proteins. However, nearly all of the genes grouped under lipid metabolism that have been previously linked to transport and secretion of lipid were regulated. For example, Apoa1 (apolipoprotein A-I) is known to be the major protein moiety of high-density lipoprotein (HDL) and is mainly produced in the liver and the intestine but is also produced in vitro by some differentiated cell lines established from human colorectal tumors [48].

We found Apoa1 (apolipoprotein A-1), Adcyap1 (adenylate cyclase activating polypeptide 1), Ltb4dh (leukotriene B4 12-hydroxydehydrogenase), Fst (follistatin), Inhbb (inhibin beta-B), Clu (clusterin), Hnf4α (hepatic nuclear factor 4, alpha), Prokr1 (prokineticin receptor 1), Pla2g1b (phospholipase A2, group IB), Sult2b1 (sulfotransferase family, cytosolic, 2B, member 1), St8sia6 (ST8 alpha-N-acetyl-neuraminide alpha-2,8-sialyltransferase 6), Cyp1b1 (cytochrome P450, family 1, subfamily b, polypeptide 1) and the Pthlh (parathyroid hormone-like peptide) to be regulated with inferred roles in lipid metabolism.

For example, Adcyap1 (adenylate cyclase activating polypeptide 1) is a member of the secretin/glucagons/vasoactive intestinal peptide (VIP) family of peptides. It has been localized by immunohistochemistry to the central nervous system, digestive tract and was shown to exhibit a variety of biological activities. It is involved in synthesis of sulfatides, glycolipid, sphingolipid and plays a role in the accumulation of estrogen and progesterone. It was also reported that Adcyap1 can regulate the proliferation and differentiation [49] and was shown to be overexpressed in neuroblastoma [50] and breast cancer [51]. Likewise, Ltb4dh (leukotriene B4 12-hydroxydehydrogenase) catalyzes the conversation of leukotriene B(4) into 12-oxo-leukotriene B(4) and is involved in reduction of 15-keto prostaglandin E1 and prostaglandin. It could be shown that Ltb4dh was overexpressed in Ta urothelial cell carcinoma [52]. We also observed Pthlh (parathyroid hormone-like peptide) to be induced in dysplasia. This protein is responsible for most cases of humoral hypercalcemia of malignancy. Pthlh expression in tumor samples has also been correlated with poor prognosis in breast cancer [53], renal carcinoma [54] and colorectal tumors [55].

Because cellular growth and differentiation is depended on highly co-ordinated transcriptional networks we also searched for master regulatory proteins of respiratory epithelium. Notably, morphogenesis and branching of respiratory epithelium depends on the timely expression and activity of transcription factors. Dynamic changes in transcriptional activation of lung-specific genes are required to get an appropriate positioning of respiratory epithelial cells with the mesenchyme-derived endothelial cells. If this critical process is disturbed, this can lead to malignancy [56]. Transcription factors involved in differentiation of pulmonary epithelium includes HNF3B (hepatocyte nuclear factor 3 beta also termed Foxa2) [57], Ttf1 (transcription termination factor, RNA polymerase I) [58], Foxf1 (forkhead box F1a) [59], Gata6 (GATA binding protein 6) [60] and Gata5 (GATA binding protein 5) [61].

In our study some of these transcription factors were highly significantly regulated in dysplasia i.e. Hnf4α, Foxa3 and Foxp2. Notably, Hnf4α (hepatocyte nuclear factor 4 alpha), a member of the steroid/thyroid hormone receptor superfamily is a transcriptional activator [62] whose ligand has been identified as tightly bound endogenous fatty acids [63]. We found Hnf4α to be highly significantly induced (12.8-fold). Hnf4α regulates constitutive expression of a large number of target genes encoding enzymes, transporters and other nuclear receptors.

Among the genes regulated by HNF4α we found at least 11 target genes up-regulated in dysplasia i.e. Arg2 (arginase type 2), Apoa1 (apoliporotein A1), Cyp1b1 (cytochrome P450, family 1, subfamily B, polypeptide 1), Cldn2 (claudin 2), Fetub (fetuin beta), Gpx2 (glutathione peroxidase 2), Gsta4 (glutathione S-transferase A4), Gpc6 (glypican 6), Hpn (hepsin), Orm1 (orosomucoid 1) and Lad1 (ladinin1) [64], therefore providing an important link between induction of this transcription factor and up-regulation of target genes.

Likewise, we found Foxa3 (forkhead box A3) to be induced 7.5-fold. This protein belongs to a group of endoderm-related developmental factors that are members of the forkhead box (Fox) superfamily of transcription factors. They were first discovered by their ability to bind to promoters of liver specific genes encoding α1-antitrypsin and transthyretin [65]. Foxa genes are regulated in early mouse embryo development [66] and are responsible, at least in part, for metabolic regulation. Mice that lack Foxa3 display an increase in the mRNA levels of various serum proteins and glycolytic enzymes and show a low serum glucagon level [67].

We also found Foxp2 (forkhead box P2) to be up-regulated 4.5-fold. This protein is a member of the new subfamily of winged-helix/forkhead DNA binding domain transcription factor. It could be shown that Foxp2 are expressed in the lung restricted to the distal epithelium and may regulate lung epithelial-specific gene transcription during embryonic development [68]. Foxp2 has been characterized as a transcriptional repressor. All the known Fox genes that were implicated in regulating lung expression were characterized as transcriptional activators [26]. This finding suggests that Foxp2 plays a role in the balance of transcriptional activation and repression that is involved in regulating epithelial cell identity and development of the lung. These processes are also crucial for the transdifferentiation of alveolar type I to type II epithelial cells, which are responsible for gas exchange and surfactant protein expression essential for lung function.

Additionally, we have confirmed the up-regulation of Areg, Ereg, Hnf4α, Foxa3 and Foxp2 by immunohistochemical staining. The immunostaining showed a distinct pattern of immunoreactivity in dysplastic cells with no staining in unaltered transgenic cells. For example, immunohistochemical staining of Areg showed a cytoplasmic immunoreactivity in dysplastic cells but not each cell expressed this molecule equally strong.

In dysplasia, however, we could not evidence altered transcriptional networks of the MAPK pathway and we did not observe regulated genes coding for cell cycle or nucleotide metabolism. Both processes are highly active in tumor cells but do not play a big role in dysplastic cells prior to malignant transformation. In this context we found two EGF ligands, Areg and Ereg to be specifically up-regulated in dysplasia. While amphiregulin binds exclusively to EGFR epiregulin binds to HER4 and mediates aberrant activation of this receptor. Indeed, exaggerated EGF tyrosine kinase activity is a probable cause for lung cancer. We now evidence de novo expression of two ligands of EGFR in dysplasia that are frequently over expressed in cancers. We did, however, not observe increased transcript expression of the cancer stem cell markers CD44, CD133 and the epithelial cell adhesion molecule EpCAM. This may support the notion that dysplasia is a facultative cancer where additional events need to occur that enable malignant transformation. Taken collectively, the whole genome expression data provided important information in the multistage process of lung cancer. Our study revealed interesting novel candidate genes and pathways that dissected the programme of respiratory epithelium from transgenic into dysplasia and eventually lung cancer. In the second part of our study we report the additional genetic events that take place from dysplasia to malignantly transformed cells and thus provide a molecular rational for the multistage process in lung cancer.

## Materials and Methods

### SP-C/c-raf model

Animals were kept according to the Public Health Service Policy on Humane Care and Use of Laboratory Animals and SP-C/c-raf transgenic mice were obtained from the laboratory of Prof. Ulf Rapp (University of Würzburg, Germany), who bred the mice in the C57BL/6/DBA/2 hybrid background. We kept the SP-C/c-raf transgenic mice in the C57BL/6 background for at least five generations.

Lung samples were derived from 5 SP-C/c-raf mice (aged 5–10 months); dysplastic and unaltered lung tissue were always isolated from the same dysplasia-bearing transgenic mouse (aged 5–7 months). Endogenous normal lung tissue was studied of 5 non-transgenic mice (aged 7–10 months). The non-transgenic littermates (wild-type) served as control for transgenic effects.

Mice were sacrificed and the lung tissues were immediately frozen on dry ice and stored at −80°C until further analysis.

The histopathological diagnosis was based on routinely processed hematoxylin-eosin stains.

### Microdissection (LMPC – Laser Microbeam Microdissection and Laser Pressure Catapulting)

From each frozen lung tissue 10-µm thick sections were prepared and transferred on polyethylene napthalate foil-covered slides (Zeiss, P.A.L.M. Microlaser Technologies GmbH, Bernried, Germany).

The sections were fixed in methanol/acetic acid and stained in hematoxylin. The desired cells were microdissected using the PALM MicroLaser systems (Zeiss, P.A.L.M. Microlaser Technologies GmbH, Bernried, Germany) and collected in an adhesive cap (Zeiss, P.A.L.M. Microlaser Technologies GmbH, Bernried, Germany). Microdissected cells were resuspended in a guanidine isothiocyanate-containing buffer (RLT buffer from RNeasy MikroKit, Qiagen, Santa Clarita, CA, USA) with 10 µl/ml β-mercaptoethanol to ensure isolation of intact RNA. Approximately an area of 6×10^6^ µm^2^ were pooled from a specific layer of interest in the same animal and used for RNA extraction.

Following microdissection, total RNA-extraction was performed with the RNeasy Micro Kit (RNeasy MicroKit Qiagen, Santa Clarita, CA, USA) according to the manufacturer's instruction. A standard quality control of the total RNA was performed using the Agilent 2100 Bioanalyzer (Agilent Technologies, Palo Alto, USA).

### cRNA labeling and hybridization to microarrays

Total RNA (median: 175 ng; range: 150–200 ng) was used to generate biotin-labeled cRNA (10 µg) by means of Message Amp aRNA Premium Amplification Kit (Ambion, Austin, TX). Quality control of cRNA was performed using a bioanalyzer (Agilent 2001 Biosizing, Agilent Technologies). Following fragmentation, labeled cRNA of each sample was hybridized to Affymetrix GeneChip® Mouse Genome 430 2.0 Arrays covering over 34.000 genes and stained according to the manufacturer's instructions.

### Quantification, normalization and statistical analysis

Array data was normalized using scaling or per-chip normalization to adjust the total or average intensity of each array to be approximately the same.

Microarray chips were analyzed by the GCOS (GeneChip Operating Software) from Affymetrix with the default settings except that the target signal was set to 500 and used to generate a microarray quality control and data report. CEL files exported from GCOS were uploaded into ArrayTrack software (National Center for Toxicological Research, U.S. FDA, Jefferson, AR, USA (NCTR/FDA)) and normalized using Total Intensity Normalization after subtracting backgrounds for data management and analysis. ArrayTrack software includes some tools common to other bioinformatics software (e.g., ANOVA, T-test and SAM).

### SAM

To compare the normalized data from dysplasia, normal lung tissue from transgenic mouse, tumor cells and non-transgenic of different mice, we used the Significance Analysis of Microarrays (SAM) algorithm (ArrayTrack), which contains a sliding scale for false discovery rate (FDR) of significantly up- and down-regulated genes [69]. All data were permuted 100 cycles by using the two classes, unpaired data mode of the algorithm. As cut-off for significance an estimated FDR of 0.001 was chosen. Moreover, a cut-off for fold-change of differential expression of 2 was used. The full description of the extraction protocol, labeling and hybridization protocol and data processing is obtainable in the GEO DATA base under http://www.ncbi.nlm.nih.gov/geo/ [accession number GSE13963].

### Principal component analysis (PCA) and hierarchical gene cluster analysis (HCA)

Two clustering approaches were used to determine components of variation in the data in this study as follows.

- Principal-component analysis (PCA) that was used to obtain a simplified visualization of entire datasets. PCA is a useful linear approach to obtain a simplified visualization of entire datasets, without losing experimental information (variance). PCA allowed the dimension of complex data to be reduced and highlights the most relevant features of a given dataset to be highlighted.
- Hierarchical gene clustering (HCA) where the data points were organized in a phylogenetic tree in which the branch lengths represent the degree of similarity between the values. HCA were conducted using the ward's minimum variance linkage clustering algorithm within ArrayTrack. After normalisation and SAM analysis a total of 2909 significant genes were used for hierarchical clustering.

### Functional analysis of the significant genes with IPA

Lists of significantly differentially expressed genes were uploaded to Ingenuity Pathways Analysis (IPA, Ingenuity Systems Inc., Redwood City, CA, USA) (www.Ingenuity.com) and functional annotation and pathway analysis was performed. IPA is a commercial, web-based interface that uses a variety of computational algorithms to identify and establish cellular networks that statistically fit the input gene list and expression values from experiments. The analysis uses a database of gene interactions culled from literature and updated every quarter of the year.

Additionally, Venn diagrams were used to examine the overlap of resulting lists of genes differentially expressed between the different sample sets.

### Quantitative real-time PCR

Corroboration of RNA expression data was performed by realtime PCR using the ABI PRISM 7500 Sequence Detection System Instrument (Applied Biosystems, Applera Deutschland GmbH, Darmstadt, Germany). Total RNA (200 ng) underwent reverse transcription using an Omniscript RT Kit (Qiagen, Santa Clarita, CA, USA) according to the manufacturer's instruction. PCR reactions were performed according to the instructions of the manufacturer using commercially available assays-on-demand (Applied Biosystems, Applera Deutschland GmbH, Darmstadt, Germany). CT values were calculated by the ABI PRISM software and relative gene expression levels were expressed as the difference in CT values of the target gene and the control gene Actin beta.

### Immunohistochemistry

Each tumor section (8 µm in thickness) was deparaffinized in roti-histol for 2 times 8 minutes, these were dehydrogenated by means of a descending alcohol row. The following incubation steps were accomplished: 2 times 3 minutes in 96% ethanol, 2 times 2 minutes in 70% Ethanol, and 2 minutes in Aqua dest. The pre-treated slices were heated in a autoclave for 15 min in citrate buffer submitted of an antigen retrieval before the colouring first. For blocking endogenous peroxidase activity the slices covered for 30 minutes with 3% hydrogen peroxide/Methanol peroxidase blocking solution. After a wash step, the slices were incubated with the primary polyclonal anti-body against AREG, EREG, HNF4α, FOXA3 and FOXP2 (Santa Cruz, Santa Cruz Biotechnologys Inc., CA, USA) for 45 minutes. After washing, a streptavidin horseradish peroxidase detection kit (Envision DAKO, Hamburg, Germany) containing 3,3′-diaminobenzidine solution as substrate was used for immunohistochemical staining according to the manufacturer's instructions. Harris Hämatoxylin was used as the counterstaining.

The specificity of the immunostaining was confirmed by negative control staining using mouse nonimmune immunoglobulin G instead of the primary antibody.

## Supporting Information

Table S1 List of genes with changed expressions that are significantly overexpressed in dysplasia versus non-altered transgenic mice: 120 significantly regulated genes. This table shows the RefSeq transcript IDs, Unigene IDs, gene titles, gene symbols, and fold changes of the significantly regulated genes.
(0.18 MB DOC)

Table S2 List of genes with changed expressions that are significantly over- or under-expressed in dysplasia versus non-transgenic mice: 287 significantly regulated genes. This table shows the RefSeq transcript IDs, Unigene IDs, gene titles, gene symbols, and fold changes of the significantly regulated genes.
(0.41 MB DOC)

Table S3 List of genes with changed expressions that are significantly over- or under-expressed in unaltered transgenic versus non-transgenic mice: 18 significantly regulated genes. This table shows the RefSeq transcript IDs, Unigene IDs, gene titles, gene symbols, and fold changes of the significantly regulated genes.
(0.04 MB DOC)

Figure S1 Ingenuity - Canonical Pathways. This figure shows the canonical pathways which were overrepresented in the group of significantly regulated genes in dysplasia versus transgenic mice.
(2.13 MB DOC)

Figure S2 Quantitative real-time PCR. Real-time PCR curves of eight genes assessed by Taqman technology as well as of the reference gene ACTB of a representative experiment are shown. The differences of the Ct values of target and ACTB (deltaCT) are indicated. The smaller the deltaCT, the higher the relative expression level of the target mRNA.
(6.75 MB DOC)

Figure S3 Quantitative real-time PCR.
(6.60 MB DOC)
