# Supplementary material for: Molecular Characterization of Lung Dysplasia Induced by c-Raf-1
Source: PLoS One. 2009 May 20;4(5):e5637. doi: 10.1371/journal.pone.0005637 (PMC2681412; doi:10.1371/journal.pone.0005637)
Supplement: Table S1 — List of genes with changed expressions that are significantly overexpressed in dysplasia versus non-altered transgenic mice: 120 significantly regulated genes. This table shows the RefSeq transcript IDs, Unigene IDs, gene titles, gene symbols, and fold changes of the significantly regulated genes. (0.18 MB DOC) [file pone.0005637.s001.doc]

| **Genename** | **Gene Title** | **Fold Change** | **Gen_id_mfr** | **RefSeq Transcript ID** |
| --- | --- | --- | --- | --- |
| Gzme | granzyme E | 47,45 | 1421227_at | NM_010373 |
| Cbln1 | cerebellin 1 precursor protein | 35,86 | 1423287_at | NM_019626 |
| Etv4 | ets variant gene 4 (E1A enhancer binding protein, E1AF) | 35,16 | 1423232_at | NM_008815 |
| 1810036H07Rik | RIKEN cDNA 1810036H07 gene | 31,07 | 1453132_a_at | NM_025467 |
| Gzme | granzyme E | 30,82 | 1450171_x_at | NM_010373 |
| 0710001A04Rik | RIKEN cDNA 0710001A04 gene | 28,84 | 1454126_at | --- |
| Apoa1 | apolipoprotein A-I | 28,06 | 1455201_x_at | NM_009692 |
| Cbln1 | cerebellin 1 precursor protein | 27,33 | 1423286_at | NM_019626 |
| Pkhd1 | polycystic kidney and hepatic disease 1 | 23,73 | 1419820_at | NM_153179 |
| Apoa1 | apolipoprotein A-I | 23,52 | 1419233_x_at | NM_009692 |
| Ndg1 /// LOC623189 | Nur77 downstream gene 1 | 21,01 | 1455423_at | NM_183322 |
| Itih2 | inter-alpha trypsin inhibitor, heavy chain 2 | 17,23 | 1417618_at | NM_010582 |
| Mcpt2 | mast cell protease 2 | 16,59 | 1449989_at | NM_008571 |
| Rgs16 | regulator of G protein signaling 16 | 16,46 | 1426037_a_at | --- |
| BC048546 | cDNA sequence BC048546 | 16,21 | 1436503_at | XM_132895 |
| Areg | amphiregulin | 15,38 | 1421134_at | NM_009704 |
| Cldn2 | claudin 2 | 13,32 | 1417231_at | NM_016675 |
| Hnf4a | hepatic nuclear factor 4, alpha | 12,81 | 1427001_s_at | NM_008261 |
| Pthlh | parathyroid hormone-like peptide | 12,48 | 1422324_a_at | NM_008970 |
| Ereg | epiregulin | 12,32 | 1419431_at | NM_007950 |
| Chl1 | cell adhesion molecule with homology to L1CAM | 12,31 | 1435190_at | NM_007697 |
| Ccdc83 | RIKEN cDNA 4932423M01 gene | 12,13 | 1453425_at | NM_029256 |
| Orm1 | orosomucoid 1 | 11,77 | 1451054_at | NM_008768 |
| St8sia6 | ST8 alpha-N-acetyl-neuramidine alpha-2,8-sialyltransferase | 11,71 | 1438566_at | --- |
| Gjb4 | gap junction membrane channel protein beta 4 | 11,14 | 1422179_at | NM_008127 |
| Cbln1 | cerebellin 1 precursor protein | 11,13 | 1423288_s_at | NM_019626 |
| Fetub | fetuin beta | 10,81 | 1449555_a_at | NM_021564 |
| Ankrd22 | ankyrin repeat domain 22 | 10,73 | 1453239_a_at | NM_024204 |
| Pbp2 | RIKEN cDNA 1700023A18 gene | 10,72 | 1424793_a_at | NM_029595 |
| Gtl2 | GTL2, imprinted maternally expressed untranslated mRNA | 10,61 | 1452183_a_at | NM_144513 |
| Rgs16 | regulator of G protein signaling 16 | 10,49 | 1455265_a_at | --- |
| Gpc6 | glypican 6 | 9,91 | 1428774_at | NM_011821 |
| 9130213B05Rik | RIKEN cDNA 9130213B05 gene | 9,75 | 1424214_at | NM_145562 |
| Apoa1 | apolipoprotein A-I | 9,75 | 1438840_x_at | NM_009692 |
| Gtl2 | GTL2, imprinted maternally expressed untranslated mRNA | 9,59 | 1428765_at | NM_144513 |
| Gja3 | gap junction protein, alpha-3 | 9,32 | 1439793_at | --- |
| Gtl2 | gene trap locus 2 | 9,27 | 1436713_s_at | --- |
| Wdr16 | RIKEN cDNA 1700019F09 gene | 9,22 | 1429552_at | NM_027963 |
| Rhbdl2 | rhomboid-like2 | 9,18 | 1442819_at | --- |
| St8sia6 | ST8 alpha-N-acetyl-neuramidine alpha-2,8-sialyltransferase | 9,13 | 1456440_s_at | --- |
| Rasgrf1 | RAS protein-specific guanine nucleotide-releasing factor 1 | 9,09 | 1435614_s_at | NM_011245 |
| Adcyap1 | adenylate cyclase activating polypeptide 1 | 8,79 | 1441778_at | NM_009625 |
| Slc35f1 | solute carrier family 35, member F1 | 8,77 | 1436719_at | NM_178675 |
| Rian | RNA imprinted and accumulated in nucleus | 8,77 | 1452899_at | --- |
| Mirg | miRNA containing gene | 8,58 | 1457030_at | XM_488655 |
| 1700027A23Rik | RIKEN cDNA 1700027A23 gene | 8,47 | 1453320_at | NM_029604 |
| Cldn4 | claudin 4 | 8,44 | 1418283_at | NM_009903 |
| Gtl2 /// Lphn1 | GTL2, imprinted maternally expressed untranslated mRNA /// latrophilin 1 | 8,27 | 1452905_at | NM_144513 |
| 9130213B05Rik | RIKEN cDNA 9130213B05 gene | 8,05 | 1428891_at | NM_145562 |
| Gtl2 | GTL2, imprinted maternally expressed untranslated mRNA | 7,93 | 1426758_s_at | NM_144513 |
| Stk39 | serine/threonine kinase 39, STE20/SPS1 homolog (yeast) | 7,89 | 1419551_s_at | NM_016866 |
| St8sia6 | ST8 alpha-N-acetyl-neuraminide alpha-2,8-sialyltransferase 6 | 7,81 | 1456147_at | NM_145838 |
| Myh6 /// LOC671894 | myosin, heavy polypeptide 6, cardiac muscle, alpha | 7,72 | 1448554_s_at | NM_010856 |
| S100a14 | S100 calcium binding protein A14 | 7,67 | 1449166_at | NM_025393 |
| 1110006E14Rik | RIKEN cDNA 1110006E14 gene | 7,65 | 1431094_at | --- |
| Atp13a4 | ATPase type 13A4 | 7,58 | 1438707_at | NM_172613 |
| Hecw1 | HECT, C2 and WW domain containing E3 ubiquitin protein ligase 1 | 7,56 | 1456527_at | XM_484217 |
| Foxa3 | forkhead box A3 | 7,45 | 1431900_a_at | NM_008260 |
| Ptprn /// LOC669060 | protein tyrosine phosphatase, receptor type, N | 7,44 | 1416588_at | NM_008985 |
| Ckmt1 | creatine kinase, mitochondrial 1, ubiquitous | 7,33 | 1417089_a_at | NM_009897 |
| Lad1 | ladinin | 7,23 | 1418449_at | NM_133664 |
| Tmem54 | RIKEN cDNA 1810017F10 gene | 7,21 | 1417895_a_at | NM_025452 |
| Brunol4 | bruno-like 4, RNA binding protein (Drosophila) | 7,19 | 1452240_at | NM_133195 |
| Cdsn | Similar to corneodesmosin precursor; S protein; differentiated keratinocyte S protein precursor | 7,17 | 1444607_at | NM_001008424 |
| D630002J15Rik | RIKEN cDNA D630002J15 gene | 7,14 | 1453480_at | XM_485742 |
| Brunol4 | bruno-like 4, RNA binding protein (Drosophila) | 7,12 | 1426930_at | NM_133195 |
| Rasgrf1 | RAS protein-specific guanine nucleotide-releasing factor 1 | 7,12 | 1422600_at | NM_011245 |
| Afp | alpha fetoprotein | 6,88 | 1416646_at | NM_007423 |
| Prss22 | protease, serine, 22 | 6,87 | 1420352_at | NM_133731 |
| LOC671894 /// LOC674761 | --- | 6,83 | 1448553_at | --- |
| Gtl2 | GTL2, imprinted maternally expressed untranslated mRNA | 6,73 | 1439380_x_at | NM_144513 |
| Cd177 | RIKEN cDNA 1190003K14 gene | 6,68 | 1424509_at | NM_026862 |
| Ptprn2 | protein-tyrosine phosphatase, receptor-type, N, polypeptide 2 | 6,60 | 1435968_at | --- |
| Ptprn2 | protein-tyrosine phosphatase, receptor-type, N, polypeptide 3 | 6,54 | 1441971_at | --- |
| Slc23a3 | solute carrier family 23 (nucleobase transporters), member 3 | 6,51 | 1460042_at | NM_194333 |
| Cyp1b1 | cytochrome P450, family 1, subfamily b, polypeptide 1 | 6,50 | 1416612_at | NM_009994 |
| Pcsk6 | proprotein convertase subtilisin/kexin type 6 | 6,42 | 1426981_at | XM_355911 |
| Sdcbp2 | syndecan binding protein (syntenin) 2 | 6,40 | 1424090_at | NM_145535 |
| Oact1 | O-acyltransferase (membrane bound) domain containing 1 | 6,33 | 1435323_a_at | NM_153546 |
| Sult2b1 | sulfotransferase family, cytosolic, 2B, member 1 | 6,30 | 1417335_at | NM_017465 |
| Afp | alpha fetoprotein | 6,27 | 1416645_a_at | NM_007423 |
| Arg2 | arginase type II | 6,25 | 1418847_at | NM_009705 |
| Gjb3 | gap junction membrane channel protein beta 3 | 6,17 | 1416715_at | NM_008126 |
| Tnfsf9 | tumor necrosis factor ligand superfamily, member 9 | 6,15 | 1422924_at | --- |
| Akr1c19 | similar to 3(20)alpha-hydroxysteroid/dihydrodiol/indanol dehydrogenase | 6,13 | 1455454_at | NM_001013785 |
| Adora1 | adenosine A1 receptor | 5,98 | 1435495_at | NM_001008533 |
| Psrc1 | RIKEN cDNA 5430413I02 gene | 5,95 | 1417323_at | NM_019976 |
| Fut2 | fucosyltransferase 2 | 5,88 | 1434862_at | NM_018876 |
| Ros1 | Ros1 proto-oncogene | 5,86 | 1425970_a_at | NM_011282 |
| Prokr1 | G protein-coupled receptor 73 | 5,82 | 1456543_at | NM_021381 |
| Ltb4dh | leukotriene B4 12-hydroxydehydrogenase | 5,71 | 1417777_at | NM_025968 |
| BC065085 | hypothetical protein A030013D21 | 5,68 | 1455872_at | NM_177628 |
| LOC675709 | UDP-Gal:betaGlcNAc beta 1,4-galactosyltransferase, polypeptide 6 | 5,55 | 1435758_at | NM_019737 |
| Gsta4 | glutathione S-transferase, alpha 4 | 5,49 | 1416368_at | NM_010357 |
| Rnf128 | ring finger protein 128 | 5,49 | 1449036_at | NM_023270 |
| Sertad4 | SERTA domain containing 4 | 5,44 | 1454877_at | NM_198247 |
| Psrc1 | RIKEN cDNA 5430413I02 gene | 5,40 | 1425416_s_at | NM_019976 |
| 4930579J09Rik | RIKEN cDNA 4930579J09 gene | 5,38 | 1418870_at | NM_133689 |
| Fst | Follistatin | 5,21 | 1434458_at | NM_008046 |
| Arg2 | arginase type II | 5,16 | 1438841_s_at | NM_009705 |
| Ly6g6c | lymphocyte antigen 6 complex, locus G6C | 5,16 | 1422749_at | NM_023463 |
| Adssl1 | adenylosuccinate synthetase like 1 | 5,14 | 1449383_at | NM_007421 |
| Hpn | hepsin | 5,08 | 1420712_a_at | NM_008281 |
| B4galt6 /// LOC675709 | UDP-Gal:betaGlcNAc beta 1,4-galactosyltransferase, polypeptide 6 | 4,85 | 1423228_at | NM_019737 |
| Tspan1 | tetraspan 1 | 4,79 | 1417957_a_at | NM_133681 |
| Alb1 | albumin 1 | 4,74 | 1425260_at | NM_009654 |
| Gpx2 | glutathione peroxidase 2 | 4,74 | 1449279_at | NM_030677 |
| Akr1b8 | aldo-keto reductase family 1, member B8 | 4,67 | 1448894_at | NM_008012 |
| Klc3 | kinesin light chain 3 | 4,66 | 1425558_at | NM_146182 |
| Cldn8 | claudin 8 | 4,60 | 1449091_at | NM_018778 |
| Foxp2 | forkhead box P2 | 4,60 | 1438232_at | NM_053242 |
| LOC675709 | UDP-Gal:betaGlcNAc beta 1,4-galactosyltransferase, polypeptide 6 | 4,50 | 1460329_at | NM_019737 |
| Krt1-18 | keratin complex 1, acidic, gene 18 | 4,49 | 1448169_at | NM_010664 |
| Clu | clusterin | 4,46 | 1418626_a_at | NM_013492 |
| Kcnk2 | potassium channel, subfamily K, member 2 | 4,34 | 1449158_at | NM_010607 |
| Pla2g1b | phospholipase A2, group IB, pancreas | 4,08 | 1416626_at | NM_011107 |
| Pcbd1 | pterin 4 alpha carbinolamine dehydratase/dimerization cofactor of hepatocyte nuclear factor 1 alpha | 4,06 | 1418713_at | NM_025273 |
| Btbd11 | BTB (POZ) domain containing 11 | 3,97 | 1428377_at | NM_001017525 |
| Golph2 | golgi phosphoprotein 2 | 3,95 | 1415698_at | NM_027307 |
| Inhbb | inhibin beta-B | 3,62 | 1426858_at | XM_148966 |
