# Supplementary material for: Molecular Characterization of Lung Dysplasia Induced by c-Raf-1
Source: PLoS One. 2009 May 20;4(5):e5637. doi: 10.1371/journal.pone.0005637 (PMC2681412; doi:10.1371/journal.pone.0005637)
Supplement: Table S2 — List of genes with changed expressions that are significantly over- or under-expressed in dysplasia versus non-transgenic mice: 287 significantly regulated genes. This table shows the RefSeq transcript IDs, Unigene IDs, gene titles, gene symbols, and fold changes of the significantly regulated genes. (0.41 MB DOC) [file pone.0005637.s002.doc]

| **Genename** | **Gene Title** | **Fold Change** | **Gen_id_mfr** | **RefSeqTranscript ID** |
| --- | --- | --- | --- | --- |
| Gzme | granzyme E | 1160,72 | 1421227_at | NM_010373 |
| Ereg | epiregulin | 482,20 | 1419431_at | NM_007950 |
| 1810036H07Rik | RIKEN cDNA 1810036H07 gene | 280,79 | 1453132_a_at | NM_025467 |
| Cbln1 | cerebellin 1 precursor protein | 239,44 | 1423287_at | NM_019626 |
| Gzme | granzyme E | 227,58 | 1450171_x_at | NM_010373 |
| Cbln1 | cerebellin 1 precursor protein | 178,86 | 1423286_at | NM_019626 |
| Gja3 | gap junction protein, alpha-3 | 151,62 | 1439793_at | --- |
| Apoa1 | apolipoprotein A-I | 148,43 | 1455201_x_at | NM_009692 |
| Cldn2 | claudin 2 | 122,38 | 1417231_at | NM_016675 |
| Mcpt2 | mast cell protease 2 | 109,92 | 1449989_at | NM_008571 |
| Apoa1 | apolipoprotein A-I | 102,84 | 1419233_x_at | NM_009692 |
| Orm1 | orosomucoid 1 | 90,18 | 1451054_at | NM_008768 |
| Dlk1 | delta-like 1 homolog (Drosophila) | 83,87 | 1449939_s_at | NM_010052 |
| Apoa1 | apolipoprotein A-I | 74,39 | 1438840_x_at | NM_009692 |
| Rhbdl2 | rhomboid-like 2 | 65,30 | 1442819_at | --- |
| Pkhd1 | polycystic kidney and hepatic disease 1 | 65,30 | 1419820_at | NM_153179 |
| 0710001A04Rik | RIKEN cDNA 0710001A04 gene | 62,95 | 1454126_at | --- |
| St8sia6 | ST8 alpha-N-acetyl-neuraminide alpha-2,8-sialyltransferase 6 | 56,45 | 1456440_s_at | --- |
| Fetub | fetuin beta | 56,15 | 1449555_a_at | NM_021564 |
| Ndg1 /// LOC623189 | Nur77 downstream gene 1 | 54,61 | 1455423_at | NM_183322 |
| Etv4 | ets variant gene 4 (E1A enhancer binding protein, E1AF) | 52,64 | 1423232_at | NM_008815 |
| Ankrd22 | ankyrin repeat domain 22 | 47,38 | 1453239_a_at | NM_024204 |
| Itih2 | inter-alpha trypsin inhibitor, heavy chain 2 | 45,75 | 1417618_at | NM_010582 |
| St8sia6 | ST8 alpha-N-acetyl-neuraminide alpha-2,8-sialyltransferase 6 | 44,18 | 1438566_at | --- |
| Rgs16 | regulator of G protein signaling 16 | 42,74 | 1426037_a_at | --- |
| St8sia6 | ST8 alpha-N-acetyl-neuraminide alpha-2,8-sialyltransferase 6 | 41,71 | 1456147_at | NM_145838 |
| Adcyap1 | adenylate cyclase activating polypeptide 1 | 41,19 | 1441778_at | NM_009625 |
| Pbp2 | RIKEN cDNA 1700023A18 gene | 37,72 | 1424793_a_at | NM_029595 |
| Cd177 | RIKEN cDNA 1190003K14 gene | 37,21 | 1424509_at | NM_026862 |
| Areg | amphiregulin | 35,63 | 1421134_at | NM_009704 |
| Gjb4 | gap junction membrane channel protein beta 4 | 35,37 | 1422179_at | NM_008127 |
| Chl1 | cell adhesion molecule with homology to L1CAM | 30,54 | 1435190_at | NM_007697 |
| Gtl2 | GTL2, imprinted maternally expressed untranslated mRNA | 30,49 | 1452183_a_at | NM_144513 |
| BC048546 | cDNA sequence BC048546 | 28,46 | 1436503_at | XM_132895 |
| Kng1 | kininogen 1 | 28,25 | 1416676_at | NM_023125 |
| 1110014F24Rik | RIKEN cDNA 1110014F24 gene | 28,13 | 1428781_at | NM_028618 |
| Rian | RNA imprinted and accumulated in nucleus | 27,14 | 1427580_a_at | --- |
| Rian | RNA imprinted and accumulated in nucleus | 27,03 | 1452899_at | --- |
| Gtl2 | GTL2, imprinted maternally expressed untranslated mRNA | 26,00 | 1428765_at | NM_144513 |
| Rasgrf1 | RAS protein-specific guanine nucleotide-releasing factor 1 | 25,78 | 1422600_at | NM_011245 |
| Atp13a4 | ATPase type 13A4 | 25,35 | 1438707_at | NM_172613 |
| Gjb3 | gap junction membrane channel protein beta 3 | 24,77 | 1416715_at | NM_008126 |
| 9130416B15 | --- | 22,98 | 1445233_at | --- |
| D630002J15Rik | RIKEN cDNA D630002J15 gene | 22,61 | 1453480_at | XM_485742 |
| Stk39 | serine/threonine kinase 39, STE20/SPS1 homolog (yeast) | 22,57 | 1419551_s_at | NM_016866 |
| Rasgrf1 | RAS protein-specific guanine nucleotide-releasing factor 1 | 22,04 | 1435614_s_at | NM_011245 |
| Fgl1 | fibrinogen-like protein 1 | 21,77 | 1424599_at | NM_145594 |
| Gtl2 /// Lphn1 | GTL2, imprinted maternally expressed untranslated mRNA /// latrophilin 1 | 19,95 | 1452905_at | NM_144513 |
| Gtl2 | GTL2, imprinted maternally expressed untranslated mRNA | 19,57 | 1426758_s_at | NM_144513 |
| Hnf4a | hepatic nuclear factor 4, alpha | 18,66 | 1427001_s_at | NM_008261 |
| Gtl2 | gene trap locus 2 | 18,61 | 1436713_s_at | --- |
| Gtl2 | GTL2, imprinted maternally expressed untranslated mRNA | 17,26 | 1439380_x_at | NM_144513 |
| Mirg | miRNA containing gene | 17,26 | 1457030_at | XM_488655 |
| Atp6v0a4 | ATPase, H+ transporting, lysosomal V0 subunit A isoform 4 | 16,92 | 1422030_at | NM_080467 |
| Tmem54 | RIKEN cDNA 1810017F10 gene | 16,56 | 1417895_a_at | NM_025452 |
| Ptprn2 | protein-tyrosine phosphatase, receptor type N, polypeptide 2 | 16,35 | 1435968_at | --- |
| Cldn4 | claudin 4 | 16,23 | 1418283_at | NM_009903 |
| Lad1 | ladinin | 16,05 | 1418449_at | NM_133664 |
| Afp | alpha fetoprotein | 15,88 | 1416645_a_at | NM_007423 |
| Slc23a3 | solute carrier family 23 (nucleobase transporters), member 3 | 15,85 | 1460042_at | NM_194333 |
| Rgs16 | regulator of G protein signaling 16 | 15,71 | 1455265_a_at | --- |
| Tnfsf9 | tumor necrosis factor ligand superfamily, member 9 | 15,68 | 1422924_at | --- |
| Afp | alpha fetoprotein | 15,07 | 1416646_at | NM_007423 |
| Prss22 | protease, serine, 22 | 14,71 | 1420352_at | NM_133731 |
| Oit1 | oncoprotein induced transcript 1 | 14,67 | 1424502_at | NM_146050 |
| 1110006E14Rik | RIKEN cDNA 1110006E14 gene | 14,51 | 1431094_at | --- |
| Rnf128 | ring finger protein 128 | 14,36 | 1449036_at | NM_023270 |
| Smurf1 /// LOC640390 | SMAD specific E3 ubiquitin protein ligase 1 | 14,16 | 1428396_at | NM_029438 |
| Pthlh | parathyroid hormone-like peptide | 14,07 | 1422324_a_at | NM_008970 |
| Sult2b1 | sulfotransferase family, cytosolic, 2B, member 1 | 13,58 | 1417335_at | NM_017465 |
| S100a14 | S100 calcium binding protein A14 | 13,42 | 1449166_at | NM_025393 |
| Ccdc83 | RIKEN cDNA 4932423M01 gene | 13,34 | 1453425_at | NM_029256 |
| Brunol4 | bruno-like 4, RNA binding protein (Drosophila) | 13,22 | 1426930_at | NM_133195 |
| Brunol4 | bruno-like 4, RNA binding protein (Drosophila) | 12,98 | 1452240_at | NM_133195 |
| Stk39 | serine/threonine kinase 39, STE20/SPS1 homolog (yeast) | 12,77 | 1419550_a_at | NM_016866 |
| Ckmt1 | creatine kinase, mitochondrial 1, ubiquitous | 12,72 | 1417089_a_at | NM_009897 |
| Cbln1 | cerebellin 1 precursor protein | 12,33 | 1423288_s_at | NM_019626 |
| Foxa3 | forkhead box A3 | 12,30 | 1431900_a_at | NM_008260 |
| BC024561 | cDNA sequence BC024561 | 11,87 | 1451610_at | NM_153576 |
| Rnf128 | ring finger protein 128 | 11,62 | 1418318_at | NM_023270 |
| Gjb1 | gap junction membrane channel protein beta 1 | 11,47 | 1448766_at | NM_008124 |
| Pglyrp1 | peptidoglycan recognition protein 1 | 11,31 | 1449184_at | NM_009402 |
| Gpc6 | glypican 6 | 11,23 | 1428774_at | NM_011821 |
| Myh6 /// LOC671894 | myosin, heavy polypeptide 6, cardiac muscle, alpha | 10,98 | 1448554_s_at | NM_010856 |
| Prokr1 | G protein-coupled receptor 73 | 10,82 | 1456543_at | NM_021381 |
| Slc26a4 | solute carrier family 26, member 4 | 10,80 | 1419725_at | NM_011867 |
| LOC671894 /// LOC674761 | --- | 10,73 | 1448553_at | --- |
| Ttc9 | tetratricopeptide repeat domain 9 | 10,64 | 1436237_at | XM_126933 |
| Ptprn /// LOC669060 | protein tyrosine phosphatase, receptor type, N | 10,59 | 1416588_at | NM_008985 |
| Ltb4dh | leukotriene B4 12-hydroxydehydrogenase | 10,49 | 1417777_at | NM_025968 |
| 9130213B05Rik | RIKEN cDNA 9130213B05 gene | 10,45 | 1424214_at | NM_145562 |
| 3110049J23Rik | RIKEN cDNA 3110049J23 gene | 10,33 | 1449462_at | NM_026085 |
| Gjb1 | gap junction membrane channel protein beta 1 | 10,18 | 1448767_s_at | NM_008124 |
| Shmt1 | serine hydroxymethyl transferase 1 (soluble) | 10,09 | 1425179_at | NM_009171 |
| LOC433393 | --- | 10,06 | 1439888_at | XM_488930 |
| Arg2 | arginase type II | 10,04 | 1418847_at | NM_009705 |
| Cdsn | Similar to corneodesmosin precursor; S protein; differentiated keratinocyte S protein precursor | 9,93 | 1444607_at | NM_001008424 |
| Cyp1b1 | cytochrome P450, family 1, subfamily b, polypeptide 1 | 9,71 | 1416612_at | NM_009994 |
| Hecw1 | HECT, C2 and WW domain containing E3 ubiquitin protein ligase 1 | 9,68 | 1456527_at | XM_484217 |
| LOC675709 | UDP-Gal:betaGlcNAc beta 1,4-galactosyltransferase, polypeptide 6 | 9,37 | 1435758_at | NM_019737 |
| Fut2 | fucosyltransferase 2 | 9,36 | 1434862_at | NM_018876 |
| 1700027A23Rik | RIKEN cDNA 1700027A23 gene | 9,28 | 1453320_at | NM_029604 |
| Psrc1 | RIKEN cDNA 5430413I02 gene | 9,18 | 1417323_at | NM_019976 |
| Ivl | RIKEN cDNA 1110019C06 gene | 9,14 | 1439878_at | --- |
| 5730559C18Rik | RIKEN cDNA 5730559C18 gene | 8,96 | 1436345_at | NM_028872 |
| Psrc1 | RIKEN cDNA 5430413I02 gene | 8,91 | 1425416_s_at | NM_019976 |
| --- | Mus musculus transcribed sequences | 8,84 | 1441971_at | --- |
| Hpn | hepsin | 8,70 | 1420712_a_at | NM_008281 |
| Adcyap1 | adenylate cyclase activating polypeptide 1 | 8,61 | 1423427_at | NM_009625 |
| Sdcbp2 | syndecan binding protein (syntenin) 2 | 8,53 | 1424090_at | NM_145535 |
| D17H6S56E-5 | DNA segment, Chr 17, human D6S56E 5 | 8,34 | 1417821_at | NM_033075 |
| Arg2 | arginase type II | 8,23 | 1438841_s_at | NM_009705 |
| Slc13a2 | solute carrier family 13 (sodium-dependent dicarboxylate transporter), member 2 | 8,07 | 1418857_at | NM_022411 |
| C430004E15Rik | RIKEN cDNA C430004E15 gene | 8,02 | 1426809_at | NM_175286 |
| Rab3c | RAB3C, member RAS oncogene family | 7,99 | 1449494_at | NM_023852 |
| E030010A14Rik | hypothetical protein E030010A14 | 7,95 | 1437595_at | NM_183160 |
| 9130213B05Rik | RIKEN cDNA 9130213B05 gene | 7,92 | 1428891_at | NM_145562 |
| Ros1 | Ros1 proto-oncogene | 7,92 | 1425970_a_at | NM_011282 |
| Syt12 | synaptotagmin 12 | 7,84 | 1422878_at | NM_134164 |
| Rap1gap | Rap1, GTPase-activating protein 1 | 7,78 | 1428443_a_at | XM_149500 |
| Plek2 | pleckstrin 2 | 7,66 | 1449424_at | NM_013738 |
| Hgfac | hepatocyte growth factor activator | 7,65 | 1418405_at | NM_019447 |
| 4931408D14Rik | RIKEN cDNA 4931408D14 gene | 7,46 | 1431806_at | --- |
| Adora1 | adenosine A1 receptor | 7,39 | 1435495_at | NM_001008533 |
| Phlda2 | pleckstrin homology-like domain, family A, member 2 | 7,35 | 1417837_at | NM_009434 |
| Slc15a2 | solute carrier family 15 (H+/peptide transporter), member 2 | 7,25 | 1424730_a_at | NM_021301 |
| Gsta4 | glutathione S-transferase, alpha 4 | 7,20 | 1416368_at | NM_010357 |
| Foxp2 | forkhead box P2 | 7,10 | 1438231_at | NM_053242 |
| Mapk13 | mitogen activated protein kinase 13 | 7,09 | 1448871_at | NM_011950 |
| Mal | myelin and lymphocyte protein, T-cell differentiation protein | 6,99 | 1417275_at | NM_010762 |
| Foxq1 | Forkhead box Q1 | 6,98 | 1438558_x_at | NM_008239 |
| Eva1 | epithelial V-like antigen 1 | 6,95 | 1416236_a_at | NM_007962 |
| Dfna5h | deafness, autosomal dominant 5 homolog (human) | 6,95 | 1417903_at | NM_018769 |
| Hdc | histidine decarboxylase | 6,94 | 1451796_s_at | NM_008230 |
| Pcbd1 | pterin 4 alpha carbinolamine dehydratase/dimerization cofactor of hepatocyte nuclear factor 1 alpha | 6,93 | 1418713_at | NM_025273 |
| Eva1 | epithelial V-like antigen 1 | 6,92 | 1448265_x_at | NM_007962 |
| Dyrk3 | dual-specificity tyrosine-(Y)-phosphorylation regulated kinase 3 | 6,92 | 1424229_at | NM_145508 |
| Adssl1 | adenylosuccinate synthetase like 1 | 6,86 | 1449383_at | NM_007421 |
| Kcnf1 | potassium voltage-gated channel, subfamily F, member 1 | 6,86 | 1454768_at | NM_201531 |
| Slc15a2 | solute carrier family 15 (H+/peptide transporter), member 2 | 6,84 | 1447808_s_at | NM_021301 |
| Foxp2 | forkhead box P2 | 6,80 | 1438232_at | NM_053242 |
| Golph2 | golgi phosphoprotein 2 | 6,79 | 1415698_at | NM_027307 |
| 4930579J09Rik | RIKEN cDNA 4930579J09 gene | 6,77 | 1418870_at | NM_133689 |
| Clu | clusterin | 6,76 | 1418626_a_at | NM_013492 |
| Akr1b8 | aldo-keto reductase family 1, member B8 | 6,76 | 1448894_at | NM_008012 |
| Wdr16 | RIKEN cDNA 1700019F09 gene | 6,75 | 1429552_at | NM_027963 |
| Mal | myelin and lymphocyte protein, T-cell differentiation protein | 6,73 | 1432558_a_at | NM_010762 |
| Wnk4 | protein kinase, lysine deficient 4 | 6,64 | 1427196_at | NM_175638 |
| B4galt6 /// LOC675709 | UDP-Gal:betaGlcNAc beta 1,4-galactosyltransferase, polypeptide 6 | 6,64 | 1423228_at | NM_019737 |
| Cldn3 | claudin 3 | 6,57 | 1426332_a_at | NM_009902 |
| Gm71 | gene model 71, (NCBI) | 6,55 | 1455726_at | XM_127052 |
| Krt1-18 | keratin complex 1, acidic, gene 18 | 6,52 | 1448169_at | NM_010664 |
| Acsl4 | acyl-CoA synthetase long-chain family member 4 | 6,44 | 1451828_a_at | NM_019477 |
| Chia | RIKEN cDNA 2200003E03 gene | 6,38 | 1416456_a_at | NM_023186 |
| Cd14 | CD14 antigen | 6,34 | 1417268_at | NM_009841 |
| LOC675709 | UDP-Gal:betaGlcNAc beta 1,4-galactosyltransferase, polypeptide 6 | 6,27 | 1460329_at | NM_019737 |
| Ace2 | angiotensin I converting enzyme (peptidyl-dipeptidase A) 2 | 6,27 | 1425102_a_at | NM_027286 |
| Cideb | cell death-inducing DNA fragmentation factor, alpha subunit-like effector B | 6,25 | 1418976_s_at | NM_009894 |
| D17H6S56E-5 | DNA segment, Chr 17, human D6S56E 5 | 6,24 | 1417822_at | NM_033075 |
| Pcsk6 | proprotein convertase subtilisin/kexin type 6 | 6,08 | 1426981_at | XM_355911 |
| Tnfrsf21 | tumor necrosis factor receptor superfamily, member 21 | 6,07 | 1450731_s_at | NM_178589 |
| Klc3 | kinesin light chain 3 | 6,04 | 1425558_at | NM_146182 |
| BC065085 | hypothetical protein A030013D21 | 6,04 | 1455872_at | NM_177628 |
| Scara3 | scavenger receptor class A, member 3 | 6,03 | 1427020_at | NM_172604 |
| Elf5 | E74-like factor 5 | 6,02 | 1419555_at | NM_010125 |
| Oact1 | O-acyltransferase (membrane bound) domain containing 1 | 6,01 | 1435323_a_at | NM_153546 |
| Ly6g6c | lymphocyte antigen 6 complex, locus G6C | 6,00 | 1422749_at | NM_023463 |
| Fgg | fibrinogen, gamma polypeptide | 5,97 | 1416025_at | NM_133862 |
| Akr1c19 | similar to 3(20)alpha-hydroxysteroid/dihydrodiol/indanol dehydrogenase | 5,95 | 1455454_at | NM_001013785 |
| Aqp3 | aquaporin 3 | 5,92 | 1450460_at | NM_016689 |
| Kcnk1 | potassium channel, subfamily K, member 1 | 5,89 | 1448690_at | NM_008430 |
| Bnipl | BCL2/adenovirus E1B 19kD interacting protein like | 5,89 | 1420683_at | NM_134253 |
| Clic6 | chloride intracellular channel 6 | 5,89 | 1454866_s_at | NM_172469 |
| 6330530A05Rik | RIKEN cDNA 6330530A05 gene | 5,88 | 1434094_at | NM_172383 |
| Sp5 | trans-acting transcription factor 5 | 5,82 | 1422914_at | NM_022435 |
| Mtac2d1 | membrane targeting (tandem) C2 domain containing 1 | 5,81 | 1439045_x_at | --- |
| Dnase2a | deoxyribonuclease II alpha | 5,75 | 1430135_at | NM_010062 |
| Crlf1 | cytokine receptor-like factor 1 | 5,70 | 1418476_at | NM_018827 |
| Tspan11 | tetraspanin 11 | 5,69 | 1430310_at | NM_026743 |
| Tspan1 | tetraspan 1 | 5,69 | 1417957_a_at | NM_133681 |
| Tspan1 | tetraspan 1 | 5,68 | 1417958_at | NM_133681 |
| Gjb2 | gap junction membrane channel protein beta 2 | 5,65 | 1423271_at | NM_008125 |
| Mt2 | metallothionein 2 | 5,62 | 1428942_at | NM_008630 |
| Hs3st3b1 | heparan sulfate (glucosamine) 3-O-sulfotransferase 3B1 | 5,62 | 1433977_at | NM_018805 |
| Kcnk2 | potassium channel, subfamily K, member 2 | 5,55 | 1449158_at | NM_010607 |
| Celsr1 | cadherin EGF LAG seven-pass G-type receptor 1 | 5,53 | 1418925_at | NM_009886 |
| Gcnt1 | glucosaminyl (N-acetyl) transferase 1, core 2 | 5,52 | 1460431_at | NM_010265 |
| Mat1a | methionine adenosyltransferase I, alpha | 5,42 | 1423147_at | NM_133653 |
| Spint1 | serine protease inhibitor, Kunitz type 1 | 5,40 | 1416627_at | NM_016907 |
| Kctd14 | potassium channel tetramerisation domain containing 14 | 5,39 | 1426633_s_at | NM_001010826 |
| Mt1 | metallothionein 1 | 5,32 | 1422557_s_at | NM_013602 |
| Psat1 | phosphoserine aminotransferase 1 | 5,32 | 1451064_a_at | NM_177420 |
| Cldn3 | claudin 3 | 5,29 | 1460569_x_at | NM_009902 |
| Foxp2 | forkhead box P2 | 5,24 | 1440108_at | NM_053242 |
| Dos | downstream of Stk11 | 5,17 | 1433494_at | XM_125771 |
| Shmt1 | serine hydroxymethyl transferase 1 (soluble) | 5,17 | 1425178_s_at | NM_009171 |
| Mod1 /// LOC624892 | malic enzyme, supernatant | 5,16 | 1430307_a_at | NM_008615 |
| Ly6g6e | lymphocyte antigen 6 complex, locus G6E | 5,14 | 1429833_at | NM_027366 |
| 2310057J16Rik | RIKEN cDNA 2310057J16 gene | 5,11 | 1432464_a_at | XM_133997 |
| Ddit4l | DNA-damage-inducible transcript 4-like | 5,11 | 1439332_at | NM_030143 |
| Tmc4 | transmembrane channel-like gene family 4 | 5,09 | 1427178_at | NM_181820 |
| Hdc | histidine decarboxylase | 5,07 | 1454713_s_at | NM_008230 |
| Sertad4 | SERTA domain containing 4 | 5,06 | 1454877_at | NM_198247 |
| Dos | downstream of Stk11 | 4,99 | 1438370_x_at | XM_125771 |
| 3300001A09Rik | RIKEN cDNA 3300001A09 gene | 4,98 | 1453326_at | XM_134869 |
| Glrx | glutaredoxin 1 (thioltransferase) | 4,94 | 1416592_at | NM_053108 |
| Cldn3 | claudin 3 | 4,94 | 1451701_x_at | NM_009902 |
| Vill | villin-like | 4,93 | 1426022_a_at | NM_011700 |
| --- | 12 days embryo spinal cord cDNA, RIKEN full-length enriched library, clone:C530045D16 product: unknown EST, full insert sequence | 4,92 | 1445501_at | --- |
| Nek6 /// LOC674247 | NIMA (never in mitosis gene a)-related expressed kinase 6 | 4,87 | 1423596_at | NM_021606 |
| Cep55 | RIKEN cDNA 1200008O12 gene | 4,84 | 1452242_at | NM_028760 |
| Azin1 | ornithine decarboxylase antizyme inhibitor | 4,82 | 1422702_at | NM_018745 |
| Shmt1 | serine hydroxymethyl transferase 1 (soluble) | 4,82 | 1425177_at | NM_009171 |
| Tacstd1 | tumor-associated calcium signal transducer 1 | 4,81 | 1416579_a_at | NM_008532 |
| Ube2c | ubiquitin-conjugating enzyme E2C | 4,78 | 1452954_at | NM_026785 |
| Spbc24 | spindle pole body component 24 homolog (S. cerevisiae) | 4,73 | 1431087_at | NM_026282 |
| Tspan11 | tetraspanin 11 | 4,69 | 1441776_at | NM_026743 |
| Cks1b | CDC28 protein kinase 1b | 4,61 | 1448441_at | NM_016904 |
| Fst | Follistatin | 4,58 | 1434458_at | NM_008046 |
| Rab25 | RAB25, member RAS oncogene family | 4,55 | 1417738_at | NM_016899 |
| Mlph | melanophilin | 4,49 | 1449896_at | NM_053015 |
| Pla2g1b | phospholipase A2, group IB, pancreas | 4,45 | 1416626_at | NM_011107 |
| Cdkn3 | cyclin-dependent kinase inhibitor 3 | 4,34 | 1430574_at | XM_484366 |
| Ttc9 | tetratricopeptide repeat domain 9 | 4,33 | 1455649_at | XM_126933 |
| St14 | suppression of tumorigenicity 14 (colon carcinoma) | 4,28 | 1418076_at | NM_011176 |
| Atp6v0a1 | ATPase, H+ transporting, lysosomal V0 subunit a isoform 1 | 4,24 | 1425227_a_at | NM_016920 |
| Gch1 | GTP cyclohydrolase 1 | 4,23 | 1420499_at | NM_008102 |
| Cblc | Casitas B-lineage lymphoma c | 4,21 | 1422666_at | NM_023224 |
| Tmem139 | RIKEN cDNA A930027H06 gene | 4,18 | 1436556_at | NM_175408 |
| Olfml2b | olfactomedin-like 2B | 4,16 | 1423915_at | NM_177068 |
| Med12l | mediator of RNA polymerase II transcription, subunit 12 homolog (yeast)-like | 4,09 | 1452864_at | --- |
| Krt2-8 | keratin complex 2, basic, gene 8 | 3,99 | 1423691_x_at | NM_031170 |
| Azin1 | ornithine decarboxylase antizyme inhibitor | 3,92 | 1450714_at | NM_018745 |
| Rbm35a | RIKEN cDNA 2210008M09 gene | 3,84 | 1454681_at | NM_194055 |
| Crispld2 | cysteine-rich secretory protein LCCL domain containing 2 | 0,20 | 1460458_at | NM_030209 |
| Centd3 | centaurin, delta 3 | 0,19 | 1419833_s_at | NM_139206 |
| BC030477 | cDNA sequence BC030477 | 0,19 | 1455845_at | NM_177618 |
| Edil3 | EGF-like repeats and discoidin I-like domains 3 | 0,19 | 1433474_at | NM_010103 |
| Acvrl1 | Activin A receptor, type II-like 1 | 0,18 | 1435825_at | NM_009612 |
| Ankrd38 | cDNA BC060737 | 0,18 | 1436425_at | NM_172872 |
| Aard | alanine and arginine rich domain containing protein | 0,17 | 1434528_at | NM_175503 |
| Centd3 | centaurin, delta 3 | 0,17 | 1451282_at | NM_139206 |
| 2210023G05Rik | RIKEN cDNA 2210023G05 gene | 0,16 | 1424968_at | NM_197999 |
| Stard9 /// LOC668856 | RIKEN cDNA 4831403C07 gene | 0,16 | 1436324_at | --- |
| 1700110N18Rik | RIKEN cDNA 1700110N18 gene | 0,16 | 1430596_s_at | XM_283372 |
| Cftr | cystic fibrosis transmembrane conductance regulator homolog | 0,16 | 1420579_s_at | NM_021050 |
| Vsnl1 | visinin-like 1 | 0,16 | 1420955_at | NM_012038 |
| Tmem100 | RIKEN cDNA 1810057C19 gene | 0,16 | 1449533_at | NM_026433 |
| LOC623121 | --- | 0,16 | 1437636_at | --- |
| 2310016C08Rik | RIKEN cDNA 2310016C08 gene | 0,16 | 1421031_a_at | NM_023516 |
| Cox4i2 | cytochrome c oxidase subunit IV isoform 2 | 0,15 | 1421373_at | NM_053091 |
| Scn3a | sodium channel, voltage-gated, type III, alpha | 0,15 | 1439204_at | XM_141275 |
| Mcc | Transcribed locus | 0,15 | 1438081_at | --- |
| Chst1 | carbohydrate (keratan sulfate Gal-6) sulfotransferase 1 | 0,15 | 1449147_at | NM_023850 |
| Upk1b | uroplakin 1b | 0,15 | 1435831_at | --- |
| Prkg2 | Protein kinase, cGMP-dependent, type II | 0,14 | 1435162_at | NM_008926 |
| Treml4 | triggering receptor expressed on myeloid cells-like 4 | 0,14 | 1460014_at | NM_172623 |
| Bmp6 | bone morphogenetic protein 6 | 0,14 | 1450759_at | NM_007556 |
| Lipg | lipase, endothelial | 0,14 | 1450188_s_at | NM_010720 |
| Cacna1d | calcium channel, voltage-dependent, L type, alpha 1D subunit | 0,14 | 1427974_s_at | NM_028981 |
| Emr4 | EGF-like module containing, mucin-like, hormone receptor-like sequence 4 | 0,14 | 1451563_at | NM_139138 |
| Cttnbp2 | cortactin binding protein 2 | 0,14 | 1435435_at | XM_289703 |
| Tcf21 | transcription factor 21 | 0,13 | 1417447_at | NM_011545 |
| G0s2 | G0/G1 switch gene | 0,13 | 1448700_at | --- |
| Wfdc6a | gene model 122, (NCBI) | 0,13 | 1457766_at | XM_130716 |
| Wnt2 | wingless-related MMTV integration site 2 | 0,13 | 1449425_at | NM_023653 |
| Adcy8 | adenylate cyclase 8 | 0,13 | 1418754_at | NM_009623 |
| Slc7a10 | solute carrier family 7 (cationic amino acid transporter, y+ system), member 10 | 0,13 | 1421093_at | NM_017394 |
| BB114106 | expressed sequence BB114106 | 0,13 | 1439527_at | --- |
| Clec14a | C-type lectin domain family 14, member a | 0,13 | 1419468_at | NM_025809 |
| Gm1337 | Gene model 1337, (NCBI) | 0,12 | 1443287_at | XM_357250 |
| Igfbp2 | insulin-like growth factor binding protein 2 | 0,12 | 1454159_a_at | NM_008342 |
| Igfbp3 | insulin-like growth factor binding protein 3 | 0,12 | 1458268_s_at | NM_008343 |
| Ptgfr | 15 days embryo head cDNA, RIKEN full-length enriched library, clone:D930037F10 | 0,12 | 1446331_at | --- |
| Gpm6a | glycoprotein m6a | 0,12 | 1456741_s_at | NM_153581 |
| AI841794 | expressed sequence AI841794 | 0,12 | 1433744_at | NM_172492 |
| Scube2 | signal peptide, CUB domain, EGF-like 2 | 0,11 | 1453486_a_at | NM_020052 |
| Fgfr4 | fibroblast growth factor receptor 4 | 0,11 | 1418596_at | --- |
| --- | Adult male olfactory brain cDNA, RIKEN full-length enriched library, clone:6430530M09 product: unclassifiable, full insert sequence | 0,10 | 1460061_at | --- |
| Lrat | Lecithin-retinol acyltransferase (phosphatidylcholine-retinol-O-acyltransferase) | 0,10 | 1444487_at | NM_023624 |
| Serpina3c | serine (or cysteine) proteinase inhibitor, clade A, member 3C | 0,10 | 1421564_at | NM_008458 |
| Chrdl1 | chordin-like1 | 0,10 | 1434201_at | --- |
| Itga8 | integrin alpha 8 | 0,10 | 1427489_at | NM_001001309 |
| 1500016O10Rik | RIKEN cDNA 1500016O10 gene | 0,09 | 1438641_x_at | XM_133706 |
| Hpcal4 | hippocalcin-like 4 | 0,09 | 1433987_at | NM_174998 |
| Igfbp3 | insulin-like growth factor binding protein 3 | 0,08 | 1423062_at | NM_008343 |
| AI481121 | expressed sequence AI481121 | 0,08 | 1456123_at | --- |
