# Supplementary material for: Molecular Characterization of Lung Dysplasia Induced by c-Raf-1
Source: PLoS One. 2009 May 20;4(5):e5637. doi: 10.1371/journal.pone.0005637 (PMC2681412; doi:10.1371/journal.pone.0005637)
Supplement: Table S3 — List of genes with changed expressions that are significantly over- or under-expressed in unaltered transgenic versus non-transgenic mice: 18 significantly regulated genes. This table shows the RefSeq transcript IDs, Unigene IDs, gene titles, gene symbols, and fold changes of the significantly regulated genes. (0.04 MB DOC) [file pone.0005637.s003.doc]

| **Genename** | **Gene Title** | **Fold Change** | **Gen_id_mfr** | **RefSeqTranscript ID** |
| --- | --- | --- | --- | --- |
| Ereg | epiregulin | 39,13 | 1419431_at | NM_007950 |
| Cyp4a12 | cytochrome P450, family 4, subfamily a, polypeptide 12 | 27,16 | 1424352_at | NM_177406 |
| Dlk1 | delta-like 1 homolog (Drosophila) | 18,61 | 1449939_s_at | NM_010052 |
| Gja3 | gap junction protein, alpha-3 | 16,27 | 1439793_at | --- |
| 1810036H07Rik | RIKEN cDNA 1810036H07 gene | 9,04 | 1453132_a_at | NM_025467 |
| P2rx2 | purinergic receptor P2X, ligand-gated ion channel, 2 | 8,37 | 1435212_at | NM_170682 |
| 1110014F24Rik | RIKEN cDNA 1110014F24 gene | 7,57 | 1428781_at | NM_028618 |
| Rhbdl2 | rhomboid-like 2 | 7,11 | 1442819_at | --- |
| St8sia6 | ST8 alpha-N-acetyl-neuraminide alpha-2,8-sialyltransferase 6 | 6,18 | 1456440_s_at | --- |
| Cd177 | RIKEN cDNA 1190003K14 gene | 5,57 | 1424509_at | NM_026862 |
| St8sia6 | ST8 alpha-N-acetyl-neuraminide alpha-2,8-sialyltransferase 6 | 5,34 | 1456147_at | --- |
| Fetub | fetuin beta | 5,19 | 1449555_a_at | NM_021564 |
| Mfsd2 | major facilitator superfamily domain containing 2 | 3,88 | 1428223_at | NM_032793 |
| Lrg1 | hypothetical protein | 3,23 | 1417290_at | NM_029796 |
| BC024561 | cDNA sequence BC024561 | 2,91 | 1451610_at | NM_153576 |
| C430004E15Rik | RIKEN cDNA C430004E15 gene | 2,28 | 1426809_at | NM_175286 |
| 9330184L24Rik | 9330184L24Rik RIKEN cDNA 9330184L24 gene | 0,12 | 1441550_at | --- |
| 5033428C03Rik | 5033428C03Rik RIKEN cDNA 5033428C03 gene | 0,08 | 1454381_at | --- |
