# Supplementary figures and images for: Molecular Characterization of Lung Dysplasia Induced by c-Raf-1
Source: PLoS One. 2009 May 20;4(5):e5637. doi: 10.1371/journal.pone.0005637 (PMC2681412; doi:10.1371/journal.pone.0005637)

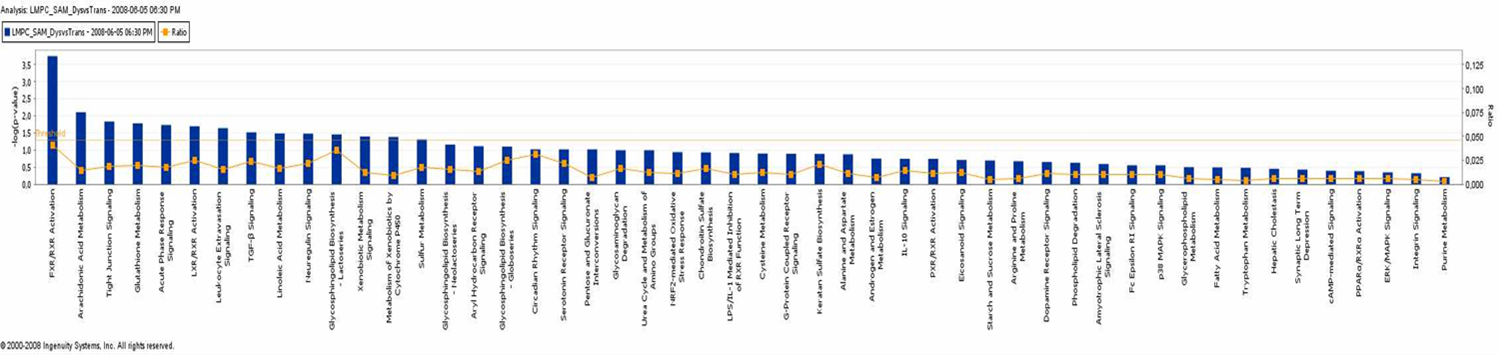

Supplement: Figure S1 — Ingenuity - Canonical Pathways. This figure shows the canonical pathways which were overrepresented in the group of significantly regulated genes in dysplasia versus transgenic mice. (2.13 MB DOC) [file pone.0005637.s004.doc]

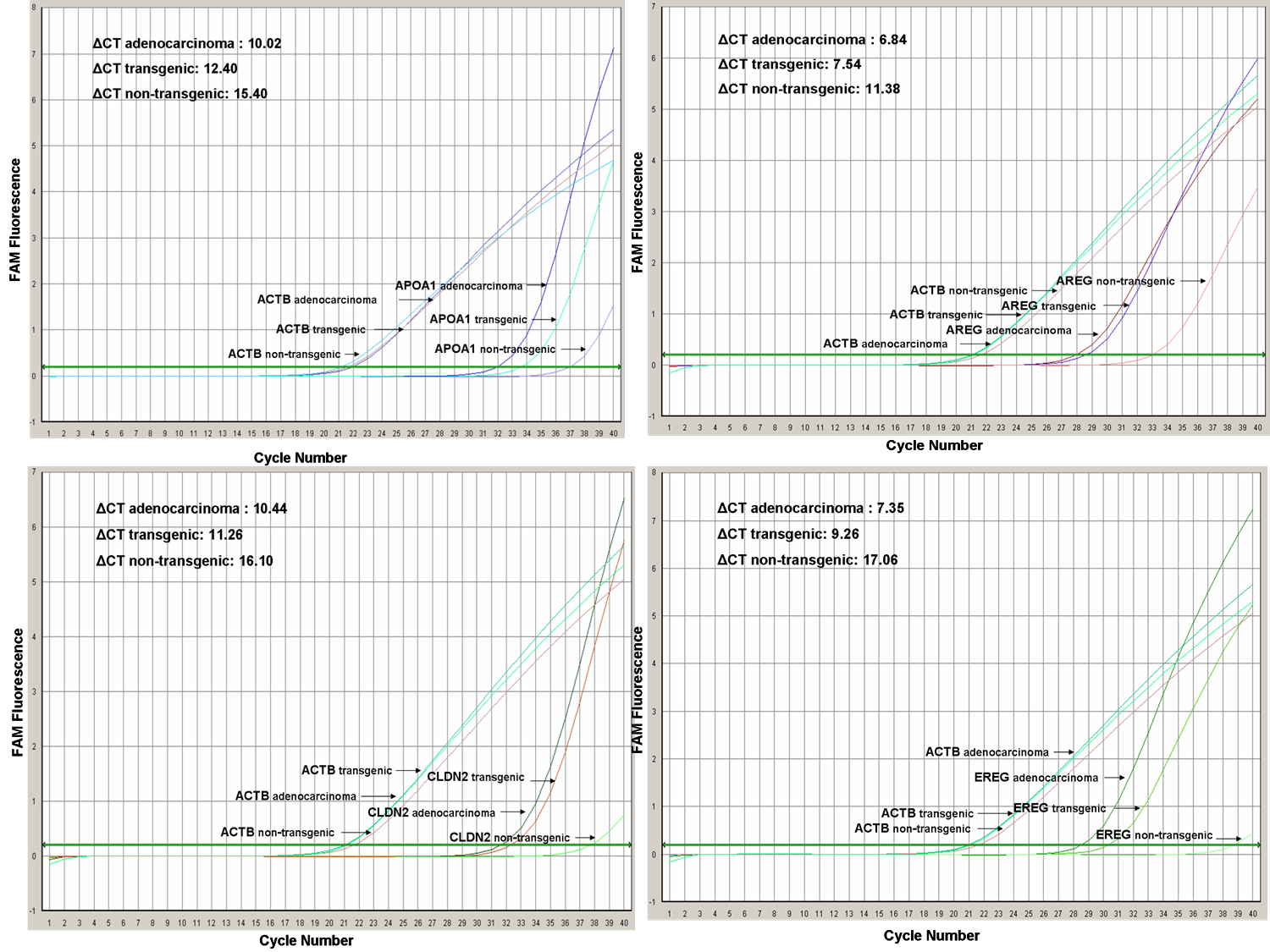

Supplement: Figure S2 — Quantitative real-time PCR. Real-time PCR curves of eight genes assessed by Taqman technology as well as of the reference gene ACTB of a representative experiment are shown. The differences of the Ct values of target and ACTB (deltaCT) are indicated. The smaller the deltaCT, the higher the relative expression level of the target mRNA. (6.75 MB DOC) [file pone.0005637.s005.doc]
